# The Adaptive designs CONSORT Extension (ACE) statement: a checklist with explanation and elaboration guideline for reporting randomised trials that use an adaptive design

**DOI:** 10.1136/bmj.m115

**Published:** 2020-06-17

**Authors:** Munyaradzi Dimairo, Philip Pallmann, James Wason, Susan Todd, Thomas Jaki, Steven A Julious, Adrian P Mander, Christopher J Weir, Franz Koenig, Marc K Walton, Jon P Nicholl, Elizabeth Coates, Katie Biggs, Toshimitsu Hamasaki, Michael A Proschan, John A Scott, Yuki Ando, Daniel Hind, Douglas G Altman

**Affiliations:** 1 School of Health and Related Research, University of Sheffield, Sheffield S1 4DA, UK; 2 Centre for Trials Research, Cardiff University, UK; 3 MRC Biostatistics Unit, University of Cambridge, UK; 4 Institute of Health and Society, Newcastle University, UK; 5 Department of Mathematics and Statistics, University of Reading, UK; 6 Department of Mathematics and Statistics, Lancaster University, UK; 7 Edinburgh Clinical Trials Unit, Usher Institute, University of Edinburgh, UK; 8 Centre for Medical Statistics, Informatics, and Intelligent Systems, Medical University of Vienna, Austria; 9 Janssen Pharmaceuticals, USA; 10 National Cerebral and Cardiovascular Center, Japan; 11 National Institute of Allergy and Infectious Diseases, National Institutes of Health, USA; 12 Division of Biostatistics in the Center for Biologics Evaluation and Research, Food and Drug Administration, USA; 13 Pharmaceuticals and Medical Devices Agency, Japan; 14 Centre for Statistics in Medicine, University of Oxford, UK

## Abstract

Adaptive designs (ADs) allow pre-planned changes to an ongoing trial without compromising the validity of conclusions and it is essential to distinguish pre-planned from unplanned changes that may also occur. The reporting of ADs in randomised trials is inconsistent and needs improving. Incompletely reported AD randomised trials are difficult to reproduce and are hard to interpret and synthesise. This consequently hampers their ability to inform practice as well as future research and contributes to research waste. Better transparency and adequate reporting will enable the potential benefits of ADs to be realised.

This extension to the Consolidated Standards Of Reporting Trials (CONSORT) 2010 statement was developed to enhance the reporting of randomised AD clinical trials. We developed an Adaptive designs CONSORT Extension (ACE) guideline through a two-stage Delphi process with input from multidisciplinary key stakeholders in clinical trials research in the public and private sectors from 21 countries, followed by a consensus meeting. Members of the CONSORT Group were involved during the development process.

The paper presents the ACE checklists for AD randomised trial reports and abstracts, as well as an explanation with examples to aid the application of the guideline. The ACE checklist comprises seven new items, nine modified items, six unchanged items for which additional explanatory text clarifies further considerations for ADs, and 20 unchanged items not requiring further explanatory text. The ACE abstract checklist has one new item, one modified item, one unchanged item with additional explanatory text for ADs, and 15 unchanged items not requiring further explanatory text.

The intention is to enhance transparency and improve reporting of AD randomised trials to improve the interpretability of their results and reproducibility of their methods, results and inference. We also hope indirectly to facilitate the much-needed knowledge transfer of innovative trial designs to maximise their potential benefits.

“To maximise the benefit to society, you need to not just do research but do it well” *Douglas G Altman*

## Purpose of the paper

Incomplete and poor reporting of randomised clinical trials makes trial findings difficult to interpret due to study methods, results, and inference that are not reproducible. This severely undermines the value of scientific research, obstructs robust evidence synthesis to inform practice and future research, and contributes to research waste.^1^ ^2^ The Consolidated Standards Of Reporting Trials (CONSORT) statement is a consensus-based reporting guidance framework that aims to promote and enhance transparent and adequate reporting of randomised trials.^3^ ^4^ Specific CONSORT extensions addressing the reporting needs for particular trial designs, hypotheses, and interventions have been developed.^5^ The use of reporting guidelines is associated with improved completeness in study reporting^6^ ^7^ ^8^; however, mechanisms to improve adherence to reporting guidelines are needed.^9^ ^10^ ^11^ ^12^

We developed an Adaptive designs CONSORT Extension (ACE)^13^ to the CONSORT 2010 statement^3^ ^4^ to support reporting of randomised trials that use an adaptive design (AD)—referred to as AD randomised trials. In this paper, we define an AD and summarise some types of ADs as well as their use and reporting. We then describe briefly how the ACE guideline was developed, and present its scope and underlying principles. Finally, we present the ACE checklist with explanation and elaboration (E&E) to guide its use.

## Adaptive designs: definition, current use, and reporting

The ACE Steering Committee^13^ agreed a definition of an AD (box 1) consistent with the literature.^14^ ^15^ ^16^ ^17^ ^18^

Box 1 **Definition of an adaptive design (AD)**

A clinical trial design that offers pre-planned opportunities to use accumulating trial data to modify aspects of an ongoing trial while preserving the validity and integrity of that trial.

Substantial uncertainties often exist when designing trials around aspects such as the target population, outcome variability, optimal treatments for testing, treatment duration, treatment intensity, outcomes to measure, and measures of treatment effect.^19^ Well designed and conducted AD trials allow researchers to address research questions more efficiently by allowing key aspects or assumptions of ongoing trials to be evaluated or validly stopping treatment arms or entire trials on the basis of available evidence.^15^ ^18^ ^20^ ^21^ As a result, patients may receive safe, effective treatments sooner than with fixed (non-adaptive) designs.^19^ ^22^ ^23^ ^24^ ^25^ Despite their potential benefits, there are practical challenges and obstacles to the use of ADs.^18^ ^26^ ^27^ ^28^ ^29^ ^30^ ^31^ ^32^ ^33^

The literature on ADs is considerable, and there is specific terminology associated with the field. Box 2 gives a glossary of key terminology used throughout this E&E document.

Box 2 **Definitions of key technical terms**

*Validity—*The ability to provide correct statistical inference to establish effects of study interventions and produce accurate estimates of effects (point estimates and uncertainty), to give results that are convincing to the broader audience (science community and consumers of research findings).
*Integrity—*Relates to minimisation of operational bias, maintenance of data confidentiality, and ensuring consistency in trial conduct (before and after adaptations) for credibility, interpretability, and persuasiveness of trial results.
*Pre-planned adaptations or adaptive features—*Pre-planned or prespecified changes or modifications to be made to aspects of an ongoing trial, which are specified at the design stage or at least before seeing accumulating trial data by treatment group, and are documented for audit trail (such as in the protocol).
*Unplanned changes—*Ad hoc modifications to aspects of an ongoing trial.
*Type of AD—*The main category used to classify a trial design by its pre-planned adaptive features or adaptations. Some ADs can fall into more than one main category of trial adaptation (see table 1).
*Adaptive decision-making criteria—*Elements of decision-making rules describing whether, how, and when the proposed trial adaptations will be used during the trial. It involves pre-specifying a set of actions guiding how decisions about implementing the trial adaptations are made given interim observed data (decision rules). It also involves pre-specifying limits or parameters to trigger trial adaptations (decision boundaries). For example, stopping boundaries that relate to pre-specified limits regarding decisions to stop the trial or treatment arm(s) early.
*Interim analysis—*A statistical analysis or review of accumulating data from an ongoing trial (interim data) to inform trial adaptations (before the final analysis), which may or may not involve treatment group comparisons.
*Binding rules—*Decision rules that must be adhered to for the design to control the false positive error rate.
*Non-binding rules—*Optional decision rules that can be overruled without negative effects on control of the false positive error rate.
*Statistical properties or operating characteristics—*Relates to behaviour of the trial design. These may include statistical power, false positive error rate, bias in estimation of treatment effect(s), or probability of each adaptation taking place.
*Simulation—*A computational procedure performed using a computer program to evaluate statistical properties of the design by generating pseudo data according to the design, under a number of scenarios and repeated a large number of times.
*Fixed (non-adaptive) design—*A clinical trial that is designed with an expected fixed sample size without any scope for pre-planned changes (adaptations) of any study design feature.
*Bias—*The systematic tendency for the treatment effect estimates to deviate from their “true values”; including the statistical properties (such as error rates) to deviate from what is expected in theory (such as pre-specified nominal error rate).
*Operational bias—*Occurs when knowledge of key trial-related information influences changes to the conduct of that trial in a manner that biases the conclusions made regarding the benefits and/or harms of study treatments.
*Statistical bias—*Bias introduced to the study results or conclusions by the design: for example, as a result of changes to aspects of the trial or multiple analyses of accumulating data from an ongoing trial.
*Subpopulation(s)—*Subset(s) of the trial population that can be classified by characteristics of participants that are thought to be associated with treatment response (such as genetic markers or biomarkers).
*Adaptation outcome(s)—*Outcome(s) used to guide trial adaptation(s); they may be different from the primary outcome(s).

Table 1 summarises some types of ADs and cites examples of their use in randomised trials. The motivations for these trial adaptations are well discussed.^15^ ^18^ ^21^ ^22^ ^25^ ^34^ ^35^ ^36^ Notably, classification of ADs in the literature is inconsistent,^13^ ^22^ while the scope and complexity of trial adaptations and underpinning statistical methods continues to broaden.^18^ ^20^ ^37^

**Table 1:** Some types of adaptations used in randomised trials with examples

Trial adaptive feature or adaptation, motivation, and cited examples of use	Type of adaptive design (AD) and examples of underlying statistical methods
Changing the predetermined sample size in response to inaccurate assumptions of study design parameters to achieve the desired statistical power.^38^-^40^	Sample size re-estimation, re-assessment, or re-calculation (SSR) using aggregated interim data from all participants or interim data separated according to allocated treatment.^41^-^48^
Stopping the trial early for efficacy, futility, or safety when there is sufficient evidence.^49^ ^50^	Group sequential design (GSD)^51^ ^52^; information-based GSD^53^; futility assessment using stochastic curtailment.^54^-^56^
Evaluating multiple treatments in one trial allowing for early selection of promising treatments or dropping futile or unsafe treatments.^57^-^59^ New treatments can also be added to an ongoing trial.^60^	Multi-arm multi-stage (MAMS), dose/treatment-selection, drop-the-loser, or pick-the-winner, or add arm.^23^ ^61^-^70^
Changing the treatment allocation ratio to favour treatments indicating beneficial effects.^71^ ^72^	Response-adaptive randomisation (RAR).^72^-^77^
Investigating multiple research objectives that are traditionally examined in distinct trial phases, in one trial under a single protocol.^78^-^80^ For instance, addressing learning (selecting promising treatments for further testing) and confirmatory objectives in one trial.	Operationally or inferentially seamless AD.^67^-^69^ ^81^-^83^
Adjusting the trial population or selecting patients with certain characteristics that are most likely to benefit from investigative treatments.^84^-^87^ This may involve incorporating statistical information from or adapting on a biomarker.	Population or patient enrichment or biomarker AD.^88^-^92^
Changing the primary research hypotheses or objectives or primary endpoints.^82^ ^93^ For example, switching from non-inferiority to superiority.	Adaptive hypotheses.^62^ ^94^
Switching the allocated treatment of patients to an alternative treatment influenced by ethical considerations, for instance, due to lack of benefit or safety issues.	Adaptive treatment-switching.^95^ ^96^
Combination of at least two types of adaptations.^24^ ^40^ ^93^ ^97^-^102^	Multiple ADs such as GSD or drop-the-loser with SSR^103^; inferentially seamless phase 2/3 AD with hypotheses selection^81^ or population enrichment^104^; biomarker-stratified with RAR^105^; adaptive platform trials where arms can be added or stopped early.^19^ ^24^ ^106^

Furthermore, there is growing literature citing AD methods^29^ ^82^ ^107^ and interest in their application by researchers and research funders.^26^ ^28^ ^108^ Regulators have published reflection and guidance papers on ADs.^14^ ^108^ ^109^ ^110^ ^111^ Several studies, including regulatory reviews, have investigated the use of ADs in randomised trials.^27^ ^29^ ^31^ ^33^ ^41^ ^49^ ^101^ ^107^ ^108^ ^112^ ^113^ ^114^ ^115^ ^116^ ^117^ ^118^ ^119^ In summary, ADs are used in a relatively low proportion of trials, although their use is steadily increasing in both the public and private sectors,^114^ ^115^ ^116^ and they are frequently considered at the design stage.^27^

The use of ADs is likely to be underestimated due to poor reporting making it difficult to retrieve them in the literature.^114^ While the reporting of standard CONSORT requirements of AD randomised trials is generally comparable to that of traditional fixed design trials,^49^ inadequate and inconsistent reporting of essential aspects relating to ADs is widely documented.^26^ ^27^ ^49^ ^107^ ^112^ ^113^ ^120^ ^121^ ^122^ This may limit their credibility, the interpretability of results, and their ability to inform or change practice,^14^ ^26^ ^27^ ^28^ ^30^ ^31^ ^108^ ^109^ ^112^ ^119^ ^120^ whereas transparency and adequate reporting can help address these concerns.^22^ ^27^ In summary, statistical and non-statistical issues arise in ADs,^22^ ^36^ ^101^ ^108^ ^123^ ^124^ ^125^ ^126^ ^127^ which require special reporting considerations.^13^

## Summary of how the ACE guideline was developed

We adhered to a registered protocol^128^ and the consensus-driven methodological framework for developing healthcare reporting guidelines recommended by the CONSORT Group and the Enhancing the QUAlity and Transparency Of health Research (EQUATOR) Network.^129^ An open access paper detailing the rationale and the complete development process of the ACE checklist for main reports and abstracts has been published.^13^ That paper details how reporting items were identified, the stakeholders who were involved, the decision-making process, consensus judgement and how reporting items were retained or dropped, and finalisation of the ACE checklist. In summary, this comprised a two-stage Delphi process involving cross-sector (public and private) and multidisciplinary key stakeholders in clinical trials research from 21 countries. Delphi survey response rates were 94/143 (66%), 114/156 (73%), and 79/143 (55%) in round one, round two, and across both rounds, respectively. A consensus meeting attended by 27 cross-sector delegates from Europe, Asia, and the US followed this. Members of the CONSORT Group provided oversight throughout. The ACE Consensus Group and Steering Committee approved the final checklist that included the abstract and contributed to this E&E document. Box 3 outlines the scope of principles guiding the application of this extension.

Box 3 **ACE guideline scope and general principles**

1. It applies to all randomised clinical trials using an adaptive design (AD), as defined in box 1.
2. It excludes randomised clinical trials that change aspects of an ongoing trial based entirely on external information^130^ or with internal pilots focusing solely on feasibility and processes (such as recruitment, intervention delivery, and data completeness).^131^
3. It covers general reporting principles to make it applicable to a wide range of current and future ADs and trial adaptations.
4. It is not intended to promote or discourage the use of any specific type of AD, trial adaptation, or frequentist or Bayesian statistical methods. These choices should be driven by the scientific research questions, the goals behind the use of the proposed AD features, and practical considerations.^22^
5. It aims to promote transparent and adequate reporting of AD randomised trials to maximise their potential benefits and improve the interpretability of their results and their reproducibility, without impeding their appropriate use or stifling design innovation. Therefore, the guideline does not specifically address the appropriateness of adaptive statistical methods.
6. It presents the minimum requirements that should be reported but we also encourage authors to report additional information that may enhance the interpretation of trial findings.
7. Access to information is most important regardless of the source and form of publication. For example, use of appendices and citation of accessible material (such as protocols, statistical analysis plans (SAPs), or related publications) is often sufficient.
8. The order in which researchers report information does not necessarily need to follow the order of the checklist.
9. The guideline does not primarily address specific reporting needs for non-randomised ADs (such as phase I dose escalation studies, phase II single-arm designs). However, some principles covered here may still apply to such trials.

## Structure of the ACE guideline

Authors should apply this guideline together with the CONSORT 2010 statement^3^ ^4^ and any other relevant extensions depending on other design features of their AD randomised trial (such as extensions for multi-arm,^132^ cluster randomised,^133^ crossover,^134^ and non-inferiority and equivalence trials^135^). Box 4 summarises the changes made to develop this extension. Table 2 shows which CONSORT 2010 items were adapted and how. We provide both CONSORT 2010 and ACE items with comments, explanation, and examples to illustrate how specific aspects of different types of AD randomised trials should be reported. For the examples, we obtained some additional information from researchers or other trial documents (such as statistical analysis plans (SAPs) and protocols). Headings of examples indicate the type of AD and the specific elements of an item that were better reported, so examples may include some incomplete reporting in relation to other elements.

Box 4 **Summary of significant changes to the CONSORT 2010 statement**

*New items—*Introduces seven new items that are specific to AD randomised trials
  - 3b on pre-planned AD features,
  - 11c on confidentiality and minimisation of operational bias,
  - 12b on estimation and inference methods,
  - 14c on adaptation decisions,
  - 15b on similarity between stages,
  - 17c on interim results and,
  - 24b on SAP and other relevant trial documents.
*Restructuring—*Renumbers four standard items to accommodate the new items
  - 3b is now 3c (on losses and exclusions) to accommodate the new item 3b,
  - 12b is now 12c (on methods for additional analyses) to accommodate the new item 12b,
  - 15 on baseline demographics and clinical characteristics is now 15a to accommodate new item 15b and,
  - 24 on access to protocol is now 24a to accommodate new item 24b.
*Modified items—*Modifies nine standard items
  - 3b (now 3c) on important changes to the design or methods after commencement,
  - 6a on pre-specified primary and secondary outcomes,
  - 6b on changes to trial outcomes after commencement,
  - 7a on sample size,
  - 7b on interim analyses and stopping rules, which is now a replacement capturing adaptive decision-making criteria to guide adaptation(s),
  - 8b on type of randomisation,
  - 12a on statistical methods to compare groups,
  - 13a on participants randomised, treated, and analysed,
  - 14a on dates for recruitment and follow-up.
*Expanded text—*Expands the E&E text for clarification on six items without changes to item wording
  - 14b on why the trial ended or was stopped,
  - 15 (now 15a) on baseline demographics and clinical characteristics,
  - 16 on numbers randomised,
  - 17a on primary and secondary outcome results,
  - 20 on limitations and,
  - 21 on generalisability.
*Restructuring—*Renames two subsection headings to reflect new ACE content
  - “recruitment” renamed to “recruitment and adaptations”
  - “sample size” renamed to “sample size and operating characteristics”
*Restructuring—*Introduces a new subsection heading
  - “Statistical analysis plan and other trial-related documents” to accommodate item 24b

**Modifies abstract item 1b and introduces an extension for journal and conference abstracts**

*New item—*Introduces one new item (on adaptation decisions made)
  On “adaptation decisions made”
*Modified item—*Modifies one standard item
  On “trial design”
*Expanded text—*Expands the E&E text for clarification on one item for certain ADs in particular circumstances without changes to item wording
  On “outcome”

Item numbers or section/topic referenced here are presented in tables 2 and 3.

**Table 2:** ACE checklist for the main report

Section/ Topic	Item No	Standard CONSORT 2010 checklist item	Extension for adaptive design randomised trials	Page No
**Title and abstract**	1a	Identification as a randomised trial in the title		
	1b	Structured summary of trial design, methods, results, and conclusions (for specific guidance see CONSORT for abstracts)^136^ ^137^	Structured summary of trial design, methods, results, and conclusions (for specific guidance see ACE for abstracts, table 3)	
**Introduction**				
Background and objectives	2a	Scientific background and explanation of rationale		
	2b	Specific objectives or hypotheses		
**Methods**				
Trial design	3a	Description of trial design (such as parallel, factorial) including allocation ratio		
	3b«^*^		Type of adaptive design used, with details of the pre-planned trial adaptations and the statistical information informing the adaptations	
	3c«3b^†^	Important changes to methods after trial commencement (such as eligibility criteria), with reasons	Important changes to the design or methods after trial commencement (such as eligibility criteria) outside the scope of the pre-planned adaptive design features, with reasons	
Participants	4a	Eligibility criteria for participants		
	4b	Settings and locations where the data were collected		
Interventions	5	The interventions for each group with sufficient details to allow replication, including how and when they were actually administered		
Outcomes	6a^†^	Completely defined pre-specified primary and secondary outcome measures, including how and when they were assessed	Completely define pre-specified primary and secondary outcome measures, including how and when they were assessed. Any other outcome measures used to inform pre-planned adaptations should be described with the rationale	
	6b^†^	Any changes to trial outcomes after the trial commenced, with reasons	Any unplanned changes to trial outcomes after the trial commenced, with reasons	
Sample size and operating characteristics	7a^†^	How sample size was determined	How sample size and operating characteristics were determined	
	7b^‡^	When applicable, explanation of any interim analyses and stopping guidelines	Pre-planned interim decision-making criteria to guide the trial adaptation process; whether decision-making criteria were binding or non-binding; pre-planned and actual timing and frequency of interim data looks to inform trial adaptations	
**Randomisation**				
Sequence generation	8a	Method used to generate the random allocation sequence		
	8b^†^	Type of randomisation; details of any restriction (such as blocking and block size)	Type of randomisation; details of any restriction (such as blocking and block size); any changes to the allocation rule after trial adaptation decisions; any pre-planned allocation rule or algorithm to update randomisation with timing and frequency of updates	
Allocation concealment mechanism	9	Mechanism used to implement the random allocation sequence (such as sequentially numbered containers), describing any steps taken to conceal the sequence until interventions were assigned		
Implementation	10	Who generated the random allocation sequence, who enrolled participants, and who assigned participants to interventions		
Blinding	11a	If done, who was blinded after assignment to interventions (for example, participants, care providers, those assessing outcomes) and how		
	11b	If relevant, description of the similarity of interventions		
	11c^*^		Measures to safeguard the confidentiality of interim information and minimise potential operational bias during the trial	
Statistical methods	12a^†^	Statistical methods used to compare groups for primary and secondary outcomes	Statistical methods used to compare groups for primary and secondary outcomes, and any other outcomes used to make pre-planned adaptations	
	12b«^*^		For the implemented adaptive design features, statistical methods used to estimate treatment effects for key endpoints and to make inferences	
	12c«12b	Methods for additional analyses, such as subgroup analyses and adjusted analyses		
**Results**				
Participant flow (a diagram is strongly recommended)	13a^†^	For each group, the numbers of participants who were randomly assigned, received intended treatment, and were analysed for the primary outcome	For each group, the numbers of participants who were randomly assigned, received intended treatment, and were analysed for the primary outcome and any other outcomes used to inform pre-planned adaptations, if applicable	
	13b	For each group, losses and exclusions after randomisation, together with reasons		
Recruitment and adaptations	14a^†^	Dates defining the periods of recruitment and follow-up	Dates defining the periods of recruitment and follow-up, for each group	
	14b^§^	Why the trial ended or was stopped	See expanded E&E text for clarification	
	14c^*^		Specify what trial adaptation decisions were made in light of the pre-planned decision-making criteria and observed accrued data	
Baseline data	15a«15^§^	A table showing baseline demographic and clinical characteristics for each group	See expanded E&E text for clarification	
	15b^*^		Summary of data to enable the assessment of similarity in the trial population between interim stages	
Numbers analysed	16^§^	For each group, number of participants (denominator) included in each analysis and whether the analysis was by original assigned groups	See expanded E&E text for clarification	
Outcomes and estimation	17a^§^	For each primary and secondary outcome, results for each group, and the estimated effect size and its precision (such as 95% confidence interval)	See expanded E&E text for clarification	
	17b	For binary outcomes, presentation of both absolute and relative effect sizes is recommended		
	17c^*^		Report interim results used to inform interim decision-making	
Ancillary analyses	18	Results of any other analyses performed, including subgroup analyses and adjusted analyses, distinguishing pre-specified from exploratory		
Harms	19	All important harms or unintended effects in each group (for specific guidance see CONSORT for harms)^138^		
**Discussion**				
Limitations	20^§^	Trial limitations, addressing sources of potential bias, imprecision, and, if relevant, multiplicity of analyses	See expanded E&E text for clarification	
Generalisability	21^§^	Generalisability (external validity, applicability) of the trial findings	See expanded E&E text for clarification	
Interpretation	22	Interpretation consistent with results, balancing benefits and harms, and considering other relevant evidence		
**Other information**				
Registration	23	Registration number and name of trial registry		
Protocol	24a«24	Where the full trial protocol can be accessed		
SAP and other relevant trial documents	24b^*^		Where the full statistical analysis plan and other relevant trial documents can be accessed	
Funding	25	Sources of funding and other support (such as supply of drugs), role of funders		

ACE = Adaptive designs CONSORT Extension. E&E = explanation and elaboration. SAP = statistical analysis plan.

“X« Y” means original CONSORT 2010 item Y has been renumbered to X;

“X«” means item reordering resulted in new item X replacing the number of the original CONSORT 2010” item X”;

* New items that should only be applied in reference to ACE;

† Modified items that require reference to both CONSORT 2010 and ACE;

‡ Replacement (modified) item that only requires reference to ACE;

§ Item wording remains unchanged in reference to CONSORT 2010 but we expanded the ACE explanatory text to clarify additional considerations for certain adaptive designs. These unchanged items require reference to CONSORT 2010 except for item 14b.

## The ACE checklist

Tables 2 and 3 are checklists for the main report and abstract, respectively. Only new and modified items are discussed in this E&E document, as well as six items that retain the CONSORT 2010^3^ ^4^ wording but require clarification for certain ADs (box 4). Authors should download and complete appendix A to accompany a manuscript during journal submission.

**Table 3:** ACE checklist for abstracts

Section/Topic	Standard checklist description	Extension for adaptive design randomised trials
Title	Identification of study as randomised	
Authors	Contact details for the corresponding author	
Trial design*	Description of the trial design (for example, parallel, cluster, non-inferiority)	Description of the trial design (for example, parallel, cluster, non-inferiority); include the word “adaptive” in the content or at least as a keyword
Methods		
Participants	Eligibility criteria for participants and the settings where the data were collected	
Interventions	Interventions intended for each group	
Objective	Specific objective or hypothesis	
Outcome†	Clearly defined primary outcome for this report	See expanded E&E text for clarification
Randomisation	How participants were allocated to interventions	
Blinding (masking)	Whether or not participants, care givers, and those assessing the outcomes were blinded to group assignment	
Results		
Numbers randomised	Number of participants randomised to each group	
Recruitment	Trial status	
Adaptation decisions made‡		Specify what trial adaptation decisions were made in light of the pre-planned decision-making criteria and observed accrued data
Numbers analysed	Number of participants analysed in each group	
Outcome	For the primary outcome, a result for each group and the estimated effect size and its precision	
Harms	Important adverse events or side effects	
Conclusions	General interpretation of the results	
Trial registration	Registration number and name of trial register	
Funding	Source of funding	

E&E = explanation and elaboration.

* Modified items that require reference to both CONSORT for abstracts^136^ ^137^ and ACE;

† Item wording remains unchanged in reference to CONSORT for abstracts,^136^ ^137^ but we expanded the ACE explanatory text to clarify additional considerations for certain adaptive designs;

‡ New items that should only be applied in reference to ACE.

### Section 1. Title and abstract

*CONSORT 2010 item 1b: Structured summary of trial design, methods, results, and conclusions (for specific guidance see CONSORT for abstracts* 136 137 *)*
*ACE item 1b: Structured summary of trial design, methods, results, and conclusions (for specific guidance see ACE for abstracts,* *table 3* *)*

*Explanation—*A well structured abstract summary encompassing trial design, methods, results, and conclusions is essential regardless of the type of design implemented.^137^ This allows readers to search for relevant studies of interest and to quickly judge if the reported trial is relevant to them for further reading. Furthermore, it helps readers to make instant judgements on key benefits and risks of study interventions. Table 3 presents minimum essential items authors should report in an AD randomised trial abstract. Authors should use this extension together with the CONSORT for journal and conference abstracts for additional details^136^ ^137^ and other relevant extensions where appropriate.

*CONSORT abstract item (Trial design): Description of the trial design (for example, parallel, cluster, non-inferiority)*
*ACE abstract item (Trial design): Description of the trial design (for example, parallel, cluster, non-inferiority); include the word “adaptive” in the content or at least as a keyword*

*Explanation—*AD randomised trials should be indexed properly to allow other researchers to easily retrieve them in literature searches. This is particularly important as trial design may influence interpretation of trial findings and the evidence synthesis approach used during meta-analyses. The MEDLINE database provides “Adaptive clinical trial” as a Medical Subject Heading (MeSH) topic to improve indexing.^139^ Authors may also like to state the type of the AD, including details of adaptations as covered under the new item 3b (table 3). See box 5 for exemplars.

Box 5 **Exemplars on the use of “adaptive” in the abstract content and/or as a keyword**

*Example 1. Abstract (title)*
  “Safety and efficacy of neublastin in painful lumbosacral radiculopathy: a randomized, double-blinded, placebo-controlled phase 2 trial using Bayesian adaptive design (the SPRINT trial).”^140^
*Example 2. Abstract (background)*
  “The drug development process can be streamlined by combining the traditionally separate stages of dose-finding (Phase IIb) and confirmation of efficacy and safety (Phase III) using an adaptive seamless design.”^141^
*Example 3. Abstract (aims) and keyword*
  “AWARD-5 was an adaptive, seamless, double-blind study comparing dulaglutide, a once-weekly glucagon-like peptide-1 (GLP-1) receptor agonist, with placebo at 26 weeks and sitagliptin up to 104 weeks.” and keyword “Bayesian adaptive”^97^

*CONSORT/ACE abstract item (Outcome): Clearly defined primary outcome for this report*

*Explanation—*In some AD randomised trials, the outcome used to inform adaptations (adaptation outcome) and the primary outcome of the study can differ (see item 6 of the main checklist for details). The necessity of reporting both of these outcomes and results in the abstract depends on the stage of reporting and whether the adaptation decisions made were critical to influencing the interpretation of the final results. For example, when a trial or at least a treatment group is stopped early, based on an adaptation outcome which is not the primary outcome, it becomes essential to adequately describe both outcomes in accordance with the CONSORT 2010 statement.^3^ ^4^ Contrarily, only the description of the primary outcome in the abstract will be essential when non-terminal adaptation decisions are made (such as to change the sample size, update randomisation, or no dropping of treatments groups at interims) and when final (not interim) results are being reported. Furthermore, the results item (table 3) should be reported consistent with the stated primary and adaptation outcome(s), where necessary. See box 6 for exemplars.

Box 6 **Exemplars on reporting outcomes in the abstract**

*Example 1. Bayesian RAR dose finding AD with early stopping for efficacy or futility*
  “The primary outcome required, first, a greater than 90% posterior probability that the most promising levocarnitine dose decreases the Sequential Organ Failure Assessment (SOFA) score at 48 hours and, second (given having met the first condition), at least a 30% predictive probability of success in reducing 28-day mortality in a subsequent traditional superiority trial to test efficacy.”^142^
*Example 2. Sequential-step AD*
  “The primary efficacy endpoint was definitive cure (absence of parasites in tissue aspirates) at 6 months. If interim analyses, based on initial cure evaluated 30 days after the start of treatment…”^143^

*ACE abstract item (adaptation decisions made): Specify what trial adaptation decisions were made in light of the pre-planned decision-making criteria and observed accrued data*

*Explanation—*A brief account of changes that were made to the trial, on what basis they were made, and when is important. The fact that the design allows for adaptations will influence interpretation of results, potentially due to operational and statistical biases. If changes should have been made, but were not, then this may further influence credibility of results. See the main checklist item 14c for details. See box 7 for exemplars.

Box 7 **Exemplars on reporting adaptation decisions made to the trial in the abstract**

*Example 1. 2-stage inferential seamless phase 2/3 AD; pre-planned adaptation decisions*
  “A planned interim analysis was conducted for otamixaban dose selection using a pre-specified algorithm (unknown to investigators) … The selected regimen to carry forward was an intravenous bolus of 0.080mg/kg followed by an infusion of 0.140 mg/kg per hour.”^144^
*Example 2. Group sequential AD; early stopping decision*
  “The trial was stopped early (at the third interim analysis), according to pre-specified rules, after a median follow-up of 27 months, because the boundary for an overwhelming benefit with LCZ696 had been crossed.”^145^

### Section 3: Methods (Trial design)

*ACE item 3b (new): Type of adaptive design used, with details of the pre-planned adaptations and the statistical information informing the adaptations*

*Explanation—*A description of the type of AD indicates the underlying design concepts and the applicable adaptive statistical methods. Although there is an inconsistent use of nomenclature to classify ADs, together with growing related methodology,^13^ some currently used types of ADs are presented in table 1. A clear description will also improve the indexing of AD methods and for easy identification during literature reviews.

Specification of pre-planned opportunities for adaptations and their scope is essential to preserve the integrity of AD randomised trials^22^ and for regulatory assessments, regardless of whether they were triggered during the trial.^14^ ^108^ ^109^ Details of pre-planned adaptations enable readers to assess the appropriateness of statistical methods used to evaluate operating characteristics of the AD (item 7a) and for performing statistical inference (item 12b). Unfortunately, pre-planned adaptations are commonly insufficiently described.^119^ Authors are encouraged to explain the scientific rationale for choosing the considered pre-planned adaptations encapsulated under the CONSORT 2010 item “scientific background and explanation of rationale” (item 2a). This rationale should focus on the goals of the considered adaptations in line with the study objectives and hypotheses (item 2b).^107^ ^108^ ^119^ ^123^

Details of pre-planned adaptations with rationale should be documented in accessible study documents for readers to be able to evaluate what was planned and unplanned (such as protocol, interim and final SAP or dedicated trial document). Of note, any pre-planned adaptation that modifies eligibility criteria (such as in population enrichment ADs^92^ ^146^) should be clearly described.

Adaptive trials use accrued statistical information to make pre-planned adaptation(s) (item 14c) at interim analyses guided by pre-planned decision-making criteria and rules (item 7b). Reporting this statistical information for guiding adaptations and how it is gathered is paramount. Analytical derivations of statistical information guiding pre-planned adaptations using statistical models or formulae should be described to facilitate reproducibility and interpretation of results. The use of supplementary material or references to published literature is sufficient. For example, sample size re-assessment (SSR) can be performed using different methods with or without knowledge or use of treatment arm allocation.^41^ ^42^ ^44^ ^48^ Around 43% (15/35) of regulatory submissions needed further clarifications because of failure to describe how a SSR would be performed.^119^ Early stopping of a trial or treatment group for futility can be evaluated based on statistical information to support lack of evidence of benefit that is derived and expressed in several ways. For example, conditional power,^56^ ^147^ ^148^ ^149^ ^150^ predictive power,^55^ ^148^ ^151^ ^152^ ^153^ the threshold of the treatment effect, posterior probability of the treatment effect,^100^ or some form of clinical utility that quantifies the balance between benefits against harms^154^ ^155^ or between patient and society perspectives on health outcomes.^100^ See box 8 for exemplars.

Box 8 **Exemplars on reporting item 3b elements**

*Example 1. Pre-planned adaptations and rationale; inferentially seamless phase 2/3 AD*
  “The adaptive (inferentially) seamless phase II/III design is a novel approach to drug development that combines phases II and III in a single, two-stage study. The design is adaptive in that the wider choice of doses included in stage 1 is narrowed down to the dose(s) of interest to be evaluated in stage 2. The trial is a seamless experience for both investigators and patients in that there is no interruption of ongoing study treatment between the two phases. Combining the dose-finding and confirmatory phases of development into a single, uninterrupted study has the advantages of speed, efficiency and flexibility^15^ ^17^… The primary aim of stage 1 of the study was to determine the risk-benefit of four doses of indacaterol (based on efficacy and safety results in a pre-planned interim analysis) in order to select two doses to carry forward into the second stage of the study.”^141^
*Example 2. Analytical derivation of statistical information to guide adaptations; population enrichment AD with SSR*
  Mehta et al^99^ detail formulae used to calculate the conditional power to guide modification of the sample size or to enrich the patient population at an interim analysis for both cutaneous and non-cutaneous patients (full population) and only cutaneous patients (subpopulation) in the supplementary material. In addition, the authors detail formulae used to derive associated conditional powers and p-values used for decision-making to claim evidence of benefit both at the interim and final analysis (linked to item 12b).
*Example 3. Pre-planned adaptations; 5-arm 2-stage AD allowing for regimen selection, early stopping for futility and SSR*
  “This randomized, placebo-controlled, double-blind, phase 2/3 trial had a two-stage adaptive design, with selection of the propranolol regimen (dose and duration) at the end of stage 1 (interim analysis) and further evaluation of the selected regimen in stage 2.^67^ ^68^ Pre-specified possible adaptations to be made after the interim analysis, as outlined in the protocol and statistical analysis plan (accessible via journal website), were selection of one or two regimens, sample-size reassessment, and non-binding stopping for futility.”^98^
*Example 4. Type of AD; pre-planned adaptations and rationale; Bayesian adaptive-enrichment AD allowing for enrichment and early stopping for futility or efficacy*
  “The DAWN trial was a multicenter, prospective, randomized, open-label trial with a Bayesian adaptive–enrichment design and with blinded assessment of endpoints.^12^ The adaptive trial design allowed for a sample size ranging from 150 to 500 patients. During interim analyses, the decision to stop or continue enrolment was based on a pre-specified calculation of the probability that thrombectomy plus standard care would be superior to standard care alone with respect to the first primary endpoint (described in the paper). The enrichment trial design gave us the flexibility to identify whether the benefit of the trial intervention was restricted to a subgroup of patients with relatively small infarct volumes at baseline. The interim analyses, which included patients with available follow-up data at the time of the analysis, were pre-specified to test for the futility, enrichment, and success of the trial.”^100^ See supplementary appendix via journal website (from page 39) for details.
*Example 5. Rationale; type of AD and pre-planned adaptations; information to inform adaptations; information-based GSD*
  “Because little was known about the variability of LVMI changes in CKD during the planning stage, we prospectively implemented an information-based (group sequential) adaptive design that allowed sample size re-estimation when 50% of the data were collected.^50^ ^156^”^157^ Pritchett et al^50^ provide details of the pre-planned adaptations and statistical information used to inform SSR and efficacy early stopping.
*Example 6. Pre-planned adaptation and information for SSR*
  “To reassess the sample size estimate, the protocol specified that a treatment-blinded interim assessment of the standard deviation (SD) about the primary endpoint (change from baseline in total exercise treadmill test duration at trough) would be performed when 231 or one half of the planned completed study patients had been randomized and followed up for 12 weeks. The recalculation of sample size, using only blinded data, was adjusted based on the estimated SD of the primary efficacy parameter (exercise duration at trough) from the aggregate data…^158^ ^159^ ^160^”^38^

*CONSORT 2010 item 3b: Important changes to the design or methods after trial commencement (such as eligibility criteria), with reasons*
*ACE item 3c (modification, renumbered): Important changes to the design or methods after trial commencement (such as eligibility criteria) outside the scope of the pre-planned adaptive design features, with reasons*

*Explanation*—Unplanned changes to certain aspects of the design or methods in response to unexpected circumstances that occur during the trial are common and will need to be reported in AD randomised trials, as in fixed design trials. This may include deviations from pre-planned adaptations and decision rules,^15^ ^70^ as well as changes to timing and frequency of interim analyses. Traditionally, unplanned changes with explanation have been documented as protocol amendments and reported as discussed in the CONSORT 2010 statement.^3^ ^4^ Unplanned changes, depending on what they are and why they were made, may introduce bias and compromise trial credibility. Some unplanned changes may render the planned adaptive statistical methods invalid or may complicate interpretation of results.^22^ It is therefore essential for authors to detail important changes that occurred outside the scope of the pre-planned adaptations and to explain why deviations from the planned adaptations were necessary. Furthermore, it should be clarified whether unplanned changes were made following access to key trial information such as interim data seen by treatment group or interim results. Such information will help readers assess potential sources of bias and implications for the interpretation of results. For ADs, it is essential to distinguish unplanned changes from pre-planned adaptations (item 3b).^161^ See box 9 for an exemplar.

Box 9 **Exemplar on reporting item 3c elements**

*Example. Inferentially seamless phase 2/3 (5-arm 2-stage) AD allowing for regimen selection, SSR and futility early stopping*
  Although this should ideally have been referenced in the main report, Léauté-Labrèze et al^98^ (on pages 17-18 of supplementary material) summarise important changes to the trial design including an explanation and discussion of implications. These changes include a reduction in the number of patients assigned to the placebo across stages—randomisation was changed from 1:1:1:1:1 to 2:2:2:2:1 (each of the 4 propranolol regimens: placebo) for stage 1 and from 1:1 to 2:1 for stage 2 in favour of the selected regimen; revised complete or nearly complete resolution success rates for certain treatment regimens. As a result, total sample size was revised to 450 (excluding possible SSR); and a slight increase in the number of patients (from 175 to 180) to be recruited for the interim analysis.

### Section 6. Outcomes

*CONSORT 2010 item 6a: Completely define pre-specified primary and secondary outcome measures, including how and when they were assessed*
*ACE item 6a (modification): Completely define pre-specified primary and secondary outcome measures, including how and when they were assessed. Any other outcome measures used to inform pre-planned adaptations should be described with the rationale*

*Comment*—Authors should also refer to the CONSORT 2010 statement^3^ ^4^ for the original text when applying this item.

*Explanation*—It is paramount to provide a detailed description of pre-specified outcomes used to assess clinical objectives including how and when they were assessed. For operational feasibility, ADs often use outcomes that can be observed quickly and easily to inform pre-planned adaptations (adaptation outcomes). Thus, in some situations, adaptations may be based on early observed outcome(s)^162^ that are believed to be informative for the primary outcome even though different from the primary outcome. The adaptation outcome (such as a surrogate, biomarker, or an intermediate outcome) together with the primary outcome influences the adaptation process, operating characteristics of the AD, and interpretation and trustworthiness of trial results. Despite many potential advantages of using early observed outcomes to adapt a trial, they pose additional risks of making misleading inferences if they are unreliable.^163^ For example, a potentially beneficial treatment could be wrongly discarded, an ineffective treatment incorrectly declared effective or wrongly carried forward for further testing, or the randomisation updated based on unreliable information.

Authors should therefore clearly describe adaptation outcomes similar to the description of pre-specified primary and secondary outcomes in the CONSORT 2010 statement.^3^ ^4^ Authors are encouraged to provide a clinical rationale supporting the use of an adaptation outcome that is different to the primary outcome in order to aid the clinical interpretation of results. For example, evidence supporting that the adaptation outcome can provide reliable information on the primary outcome will suffice. See box 10 for exemplars.

Box 10 **Exemplars on reporting item 6a elements**

*Example 1. SSR; description of the adaptation and primary outcomes*
  “The primary endpoint is a composite of survival free of debilitating stroke (modified Rankin score >3) or the need for a pump exchange. The short-term endpoint will be assessed at 6 months and the long-term endpoint at 24 months (primary). Patients who are urgently transplanted due to a device complication before a pre-specified endpoint will be considered study failures. All other transplants or device explants due to myocardial recovery that occur before a pre-specified endpoint will be considered study successes ... The adaptation was based on interim short-term outcome rates.”^164^
*Example 2. Seamless phase 2/3 Bayesian AD with treatment selection; details of adaptation outcomes*
  “Four efficacy and safety measures were considered important for dose selection based on early phase dulaglutide data: HbA1c, weight, pulse rate and diastolic blood pressure (DBP).^165^ These measures were used to define criteria for dose selection. The selected dulaglutide dose(s) had to have a mean change of ≤+5 beats per minute (bpm) for PR and ≤+2 mmHg for DBP relative to placebo at 26 weeks. In addition, if a dose was weight neutral versus placebo, it had to show HbA1c reduction ≥1.0% and/or be superior to sitagliptin at 52 weeks. If a dose reduced weight relative to placebo ≥2.5 kg, then non-inferiority to sitagliptin would be acceptable. A clinical utility index was incorporated in the algorithm to facilitate adaptive randomization and dose selection^154^ ^166^ based on the same parameters used to define dose-selection criteria described above (not shown here).”^97^
*Example 3. Seamless phase 2/3 AD with treatment selection; details of adaptation outcomes*
  “For the dose selection, the joint primary efficacy outcomes were the trough FEV_1_ on Day 15 (mean of measurements at 23 h 10 min and 23 h 45 min after the morning dose on Day 14) and standardized (average) FEV_1_ area under the curve (AUC) between 1 and 4 h after the morning dose on Day 14 (FEV_1_AUC_1–4h_), for the treatment comparisons detailed below (not shown here).”^141^
*Example 4. MAMS AD; adaptation rationale (part of item 3b); rationale for adaption outcome different from the primary outcome; description of the adaptation and primary outcomes*
  “This seamless phase 2/3 design starts with several trial arms and uses an intermediate outcome to adaptively focus accrual away from the less encouraging research arms, continuing accrual only with the more active interventions. The definitive primary outcome of the STAMPEDE trial is overall survival (defined as time from randomisation to death from any cause). The intermediate primary outcome is failure-free survival (FFS) defined as the first of: PSA failure (PSA >4 ng/mL and PSA >50% above nadir); local progression; nodal progression; progression of existing metastases or development of new metastases; or death from prostate cancer. FFS is used as a screening method for activity on the assumption that any treatment that shows an advantage in overall survival will probably show an advantage in FFS beforehand, and that a survival advantage is unlikely if an advantage in FFS is not seen. Therefore, FFS can be used to triage treatments that are unlikely to be of sufficient benefit. It is not assumed that FFS is a surrogate for overall survival; an advantage in FFS might not necessarily translate into a survival advantage.”^167^

*CONSORT 2010 item 6b: Any changes to trial outcomes after the trial commenced, with reasons*
*ACE item 6b (modification): Any unplanned changes to trial outcomes after the trial commenced, with reasons*

*Comment*—Authors may wish to cross-reference the CONSORT 2010 statement^3^ ^4^ for background details.

*Explanation*—Outcome reporting bias occurs when the selection of outcomes to report is influenced by the nature and direction of results. The prevalence of outcome reporting bias in medical research is well documented: discrepancies between pre-specified outcomes in protocols or registries and those published in reports^12^ ^168^ ^169^ ^170^ ^171^; outcomes that portray favourable beneficial effects of treatments and safety profiles being more likely to be reported^169^; some pre-specified primary or secondary outcomes modified or switched after trial commencement.^170^ Changes to trial outcomes may also include changes to how outcomes were assessed or measured, when they were assessed, or the order of importance to address objectives.^171^

Sometimes when planning trials, there is huge uncertainty around the magnitude of treatment effects on potential outcomes viewed acceptable as primary endpoints.^36^ ^171^ As a result, although uncommon, a pre-planned adaptation could include the choice of the primary endpoints or hypotheses for assessing the benefit-risk ratio. In such circumstances, the adaptive strategy should be clearly described as a pre-planned adaptation (item 3b). Authors should clearly report any additional changes to outcomes outside the scope of the pre-specified adaptations including an explanation of why such changes occurred in line with the CONSORT 2010 statement. This will enable readers to distinguish pre-planned trial adaptations of outcomes from unplanned changes, thereby allowing them to judge outcome reporting bias. See box 11 for an exemplar.

Box 11 **Exemplar on reporting item 6b**

*Example. Bayesian adaptive-enrichment AD; unplanned change from a secondary to a co-primary outcome, rationale, and when it happened*
  “The second primary endpoint was the rate of functional independence (defined as a score of 0, 1, or 2 on the modified Rankin scale) at 90 days. This endpoint was changed from a secondary endpoint to a co-primary endpoint at the request of the Food and Drug Administration at 30 months after the start of the trial, when the trial was still blinded.”^100^

### Section 7. Sample size and operating characteristics

*CONSORT 2010 item 7a: How sample size was determined*
*ACE item 7a (modification): How sample size and operating characteristics were determined*

*Comments*—This section heading was modified to reflect additional operating characteristics that may be required for some ADs in addition to the sample size. Items 3b, 7a, 7b, and 12b are connected so they should be cross-referenced when reporting.

*Explanation*—Operating characteristics, which relate to the statistical behaviour of a design, should be tailored to address trial objectives and hypotheses, factoring in logistical, ethical, and clinical considerations. These may encompass the maximum sample size, expected sample sizes under certain scenarios, probabilities of identifying beneficial treatments if they exist, and probabilities of making false positive claims of evidence.^172^ ^173^ Specifically, the predetermined sample size for ADs is influenced, among other things, by:

1. Type and scope of adaptations considered (item 3b);
2. Decision-making criteria used to inform adaptations (item 7b);
3. Criteria for claiming overall evidence (such as based on the probability of the treatment effect being above a certain value, targeted treatment effect of interest, and threshold for statistical significance^174^ ^175^);
4. Timing and frequency of the adaptations (item 7b);
5. Type of primary outcome(s) (item 6a) and nuisance parameters (such as outcome variance);
6. Method for claiming evidence on multiple key hypotheses (part of item 12b);
7. Desired operating characteristics (see box 2), such as statistical power and an acceptable level of making a false positive claim of benefit;
8. Adaptive statistical methods used for analysis (item 12b);
9. Statistical framework (frequentist or Bayesian) used to design and analyse the trial.

Information that guided estimation of sample size(s), including operating characteristics of the considered AD, should be described sufficiently to enable readers to reproduce the sample size calculation. The assumptions made concerning design parameters should be clearly stated and supported with evidence if possible. Any constraints imposed (for example, due to limited trial population) should be stated. It is good scientific practice to reference the statistical tools used (such as statistical software, program, or code) and to describe the use of statistical simulations when relevant (see item 24b discussion).

In a situation where changing the sample size is a pre-planned adaptation (item 3b), authors should report the initial sample sizes (at interim analyses before the expected change in sample size) and the maximum allowable sample size per group and in total if applicable. The planned sample sizes (or expected numbers of events for time-to-event data) at each interim analysis and final analysis should be reported by treatment group and overall. The timing of interim analyses can be specified as a fraction of information gathered rather than sample size. See box 12 for exemplars.

Box 12 **Exemplars on reporting item 7a elements**

**Fig 1:**
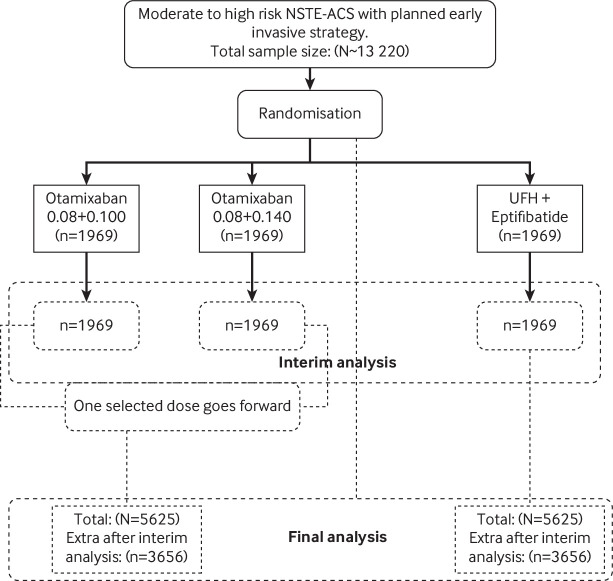
Adapted from Steg et al.^197^

*Example 1. MAMS AD; assumptions and adaptive methods; approach for claiming evidence or informing adaptations; statistical program*
  “The primary response (outcome) from each patient is the difference between the baseline HOMA-IR score and their HOMA-IR score at 24 weeks. The sample size calculation is based on a one-sided type I error of 5% and a power of 90%. If there is no difference between the mean response on any treatment and that on control, then a probability of 0.05 is set for the risk of erroneously ending the study with a recommendation that any treatment be tested further. For the power, we adopt a generalisation of this power requirement to multiple active treatments due to Dunnett.^176^ Effect sizes are specified as the percentage chance of a patient on active treatment achieving a greater reduction in HOMA-IR score than a patient on control as this specification does not require knowledge of the common SD, σ. The requirement is that, if a patient on the best active dose has a 65% chance of a better response than a patient on control, while patients on the other two active treatments have a 55% chance of showing a better response than a patient on control, then the best active dose should be recommended for further testing with 90% probability. A 55% chance of achieving a better response on active dose relative to control corresponds to a reduction in mean HOMA-IR score of about a sixth of an SD (0.178σ), while the clinically relevant effect of 65% corresponds to a reduction of about half an SD (0.545σ). The critical values for recommending that a treatment is taken to further testing at the interim and final analyses (2.782 and 2.086) have been chosen to guarantee these properties using a method described by Magirr et al,^177^ generalising the approach of Whitehead and Jaki.^178^ The maximum sample size of this study is 336 evaluable patients (84 per arm), although the use of the interim analysis may change the required sample size. The study will recruit additional patients to account for an anticipated 10% dropout rate (giving a total sample size of 370). An interim analysis will take place once the primary endpoint is available for at least 42 patients on each arm (i.e., total of 168, half of the planned maximum of 336 patients). Sample size calculation was performed using the MAMS package in R^179^.”^57^
*Example 2. 3-arm 2-stage AD with dose selection; group sequential approach; assumptions; adaptation decision-making criteria; stage 1 and 2 sample sizes; use of simulations*
  “Sample size calculations are based on the primary efficacy variable (composite of all-cause death or new MI through day 7), with the following assumptions: an event rate in the control group of 5.0%, based on event rates from the phase II study (24); a relative risk reduction (RRR) of 25%; a binomial 1-sided (α=0.025) superiority test for the comparison of 2 proportions with 88% power; and a 2-stage adaptive design with one interim analysis at the end of stage 1 data (35% information fraction) to select 1 otamixaban dose for continuation of the study at stage 2. Selection of the dose for continuation was based on the composite end point of all-cause death, Myocardial Infarction (MI), thrombotic complication, and the composite of Thrombosis in Myocardial Infarction (TIMI) major bleeding through day 7, with an assumed probability for selecting the “best” dose according to the primary endpoint (r=0.6), a group sequential approach with futility boundary of relative risk of otamixaban versus UFH plus eptifibatide ≥1.0, and efficacy boundary based on agamma (−10) α spending function.^180^ Based on the above assumptions, simulations (part of *item 24b*, see supplementary material) showed that 13 220 patients (a total of 5625 per group for the 2 remaining arms for the final analysis) are needed for this study.”^181^ See figure 1.

*CONSORT 2010 item 7b: When applicable, explanation of any interim analyses and stopping guidelines*
*ACE item 7b (replacement): Pre-planned interim decision-making criteria to guide the trial adaptation process; whether decision-making criteria were binding or non-binding; pre-planned and actual timing and frequency of interim data looks to inform trial adaptations*

*Comments*—This item is a replacement so when reporting, the CONSORT 2010^3^ item 7b content should be ignored. Items 7b and 8b overlap, but we intentionally reserved item 8b specifically to enhance complete reporting of ADs with randomisation updates as a pre-planned adaptation. Reporting of these items is also connected to items 3b and 12b.

*Explanation*—Transparency and complete reporting of pre-planned decision-making criteria (box 2) and how overall evidence is claimed are essential as they influence operating characteristics of the AD, credibility of the trial, and clinical interpretation of findings.^22^ ^32^ ^182^

A key feature of an AD is that interim decisions about the course of the trial are informed by observed interim data (element of item 3b) at one or more interim analyses guided by decision rules describing how and when the proposed adaptations will be activated (pre-planned adaptive decision-making criteria). Decision rules, as defined in box 2, may include, but are not limited to, rules for making adaptations described in table 1. Decision rules are often constructed with input of key stakeholders (such as clinical investigators, statisticians, patient groups, health economists, and regulators).^183^ For example, statistical methods for formulating early stopping decision rules of a trial or treatment group(s) exist.^51^ ^52^ ^184^ ^185^ ^186^ ^187^

Decision boundaries (for example, stopping boundaries), pre-specified limits or parameters used to determine adaptations to be made, and criteria for claiming overall evidence of benefit and/or harm (at an interim or final analysis) should be clearly stated. These are influenced by statistical information used to inform adaptations (item 3b). Decision trees or algorithms can aid the representation of complex adaptive decision-making criteria.

Allowing for trial adaptations too early in a trial with inadequate information severely undermines robustness of adaptive decision-making criteria and trustworthiness of trial results.^188^ ^189^ Furthermore, methods and results can only be reproducible when timing and frequency of interim analyses are adequately described. Therefore, authors should detail when and how often the interim analyses were planned to be implemented. The planned timing can be described in terms of information such as interim sample size or number of events relative to the maximum sample size or maximum number of events, respectively. For example, in circumstances when the pre-planned and actual timing or/and frequency of the interim analyses differ, reports should clearly state what actually happened (item 3c).

Clarification should be made on whether decision rules were binding or non-binding to help assess implications in the case when they were overruled or ignored. For example, when a binding futility boundary is overruled and a trial is continued, this would lead to a type I error inflation. Non-binding decision rules are those that can be overruled without having a negative effect on the control of the type I error rate. Use of non-binding futility boundaries is often advised.^55^ See box 13 for exemplars.

Box 13 **Exemplars on reporting item 7b elements**

*Example 1. 2-arm 2-stage AD with options for early stopping for futility or superiority and to increase the sample size; binding stopping rules*
  “To calculate the number of patients needed to meet the primary endpoint, we expected a 3-year overall survival rate of 25% in the group assigned to preoperative chemotherapy (arm A) (based on two previous trials^190^ ^191^). In comparison, an increase of 10% (up to 35%) was anticipated by preoperative CRT. Using the log-rank test (one-sided at this point) at a significance level of 5%, we calculated to include 197 patients per group to ensure a power of 80%. In the first stage of the planned two-stage adaptive design,^192^ the study was planned to be continued on the basis of a new calculation of patients needed if the comparison of patient groups will be 0.0233< p_1_< 0.5. Otherwise, the study may be closed for superiority (p_1_< 0.0233) or shall be closed for futility (p_1_≥ 0.5). There was no maximum sample size cap and stopping rules were binding.”^193^ Values p_1_ and p_2_ are p-values derived from independent stage 1 and stage 2 data, respectively. Evidence of benefit will be claimed if the overall two-stage p-value derived from p_1_ and p_2_ is ≤0.05.
*Example 2. Timing and frequency of interim analyses; planned stopping boundaries for superiority and futility*. See table 4

**Table 4:** Stopping boundaries

Interim analysis	Number of primary outcome events (information fraction)	Stopping boundaries				
		Superiority			Futility	
		Hazard ratio	P-value		Hazard ratio	P-value
1	800 (50%)	<0.768	<0.0002		>0.979	>0.758
2	1200 (75%)	<0.806	<0.0002		>0.931	>0.216
Final	1600 (100%)	<0.906	<0.0500			

Adapted from Pocock et al^194^; primary outcome events are cardiovascular deaths, myocardial infarction, or ischaemic stroke.

*Example 3. Planned timing and frequency of interim analyses; pre-specified dose selection rules for an inferentially seamless phase 2/3 (7-arm 2-stage) AD*
  “The interim analysis was pre-planned for when at least 110 patients per group (770 total) had completed at least 2 weeks of treatment. The dose selection guidelines were based on efficacy and safety. The mean effect of each indacaterol dose versus placebo was judged against pre-set efficacy reference criteria for trough FEV_1_ and FEV_1_AUC_1–4h_. For trough FEV_1_, the reference efficacy criterion was the highest value of: (a) the difference between tiotropium and placebo, (b) the difference between formoterol and placebo, or (c) 120 mL (regarded as the minimum clinically important difference). For standardized FEV_1_AUC_1–4h_, the reference efficacy criterion was the highest value of: (a) the difference between tiotropium and placebo or (b) the difference between formoterol and placebo. If more than one indacaterol dose exceeded both the efficacy criteria, the lowest effective dose plus the next higher dose were to be selected. Data on peak FEV_1_, % change in FEV_1_, and FVC were also supplied to the DMC for possible consideration, but these measures were not part of the formal dose selection process and are not presented here. The DMC also took into consideration any safety signals observed in any treatment arm.”^141^
*Example 4. Timing and frequency of interim analyses; decision-making criteria for population enrichment and sample size increase*
  “Cohort 1 will enrol a total of 120 patients and followed them until 60 PFS events are obtained. At an interim analysis based on the first 40 PFS events, an independent data monitoring committee will compare the conditional power for the full population (CP_F_) and the conditional power for the cutaneous subpopulation (CP_S_). The formulae for these conditional powers are given in the supplementary appendix (*part of item 3b, example 2,* *box 8*). (a) If CP_F_ <0.3 and CP_S_ <0.5, the results are in the unfavourable zone; the trial will enrol 70 patients to cohort 2 and follow them until 35 PFS events are obtained (then test effect in the full population). (b) If CP_F_ <0.3 and CP_S_ >0.5, the results are in the enrichment zone; the trial will enrol 160 patients with cutaneous disease (subpopulation) to cohort 2 and follow them until 110 PFS events have been obtained from the combined patients in both cohorts with cutaneous disease only (then test effect only in the cutaneous subpopulation). (c) If 0.3≤ CP_F_ ≤0.95, the results are in the promising zone (so increase sample size); the trial will enrol 220 patients (full population) to cohort 2 and follow them up until 110 PFS events are obtained (then test effect in the full population). (d) If CP_F_ >0.95, the results are in the favourable zone; the trial will enrol 70 patients to cohort 2 and follow them until 35 PFS events are obtained (then test effect in full population).”^99^ See figure 2 of Mehta et al^99^ for a decision-making tree.
*Example 5. Bayesian GSD with futility early stopping; frequency and timing of interim analyses; adaptation decision-making criteria; criteria for claiming treatment benefit*
  “We adopted a group-sequential Bayesian design^197^ with three stages, of 40 patients each (in total), and two interim analyses after 40 and 80 randomised participants, and a final analysis after a maximum of 120 randomised participants. We decided that the trial should be stopped early if there is a high (posterior) probability (90% or greater) (*item 3b* details) that the 90-day survival odds ratio (OR) falls below 1 (i.e. REBOA is harmful) at the first or second interim analysis. REBOA will be declared “successful” if the probability that the 90-day survival OR exceeds 1 at the final analysis is 95% or greater.”^195^

Additional examples on the use of non-binding futility boundaries and a cap on sample size following SSR and treatment selection are given in Appendix B.

### Section 8. Randomisation (Sequence generation)

*CONSORT 2010 item 8b: Type of randomisation; details of any restriction (such as blocking and block size)*
*ACE item 8b (modification): Type of randomisation; details of any restriction (such as blocking and block size); any changes to the allocation rule after trial adaptation decisions; any pre-planned allocation rule or algorithm to update randomisation with timing and frequency of updates*

*Comments—*In applying this item, the reporting of randomisation aspects before activation of trial adaptations must adhere to CONSORT 2010 items 8a and 8b. This E&E document only addresses additional randomisation aspects that are essential when reporting any AD where the randomisation allocation changes. Note that the contents of extension items 7b and 8b overlap.

*Explanation*—In AD randomised trials, the allocation ratio(s) may remain fixed throughout or change during the trial as a consequence of pre-planned adaptations (for example, when modifying randomisation to favour treatments more likely to show benefits, after treatment selection, or upon introduction of a new arm to an ongoing trial).^73^ Unplanned changes may also change allocation ratios (for example, after early stopping of a treatment arm due to unforeseeable harms).

This reporting item is particularly important for response-adaptive randomisation (RAR) ADs as several factors influence their efficiency and operating characteristics, which in turn influence the trustworthiness of results and necessitate adequate reporting.^13^ ^196^ ^197^ ^198^ ^199^ For RAR ADs, authors should therefore detail the pre-planned:

- Burn-in period before activating randomisation updates, including the period when the control group allocation ratio was fixed;
- Type of randomisation method with allocation ratios per group during the burn-in period as detailed in the standard CONSORT 2010 item 8b;
- Method or algorithm used to adapt or modify the randomisation allocations after the burn-in period;
- Information used to inform the adaptive randomisation algorithm and how it was derived (item 3b). Specifically, when a Bayesian RAR is used, we encourage authors to provide details of statistical models and rationale for the prior distribution chosen;
- Frequency of updating the allocation ratio (for example, after accrual of a certain number of participants with outcome data or defined regular time period) and;
- Adaptive decision-making criteria to declare early evidence in favour or against certain treatment groups (part of item 7b).

In addition, any envisaged changes to the allocation ratio as a consequence of other trial adaptations (for example, early stopping of an arm or addition of a new arm) should be stated. See box 14 for exemplars.

Box 14 **Exemplars on reporting item 8b elements**

*Example 1. Pre-planned changes to allocation ratios as a consequence of treatment selection or/and sample size increase*
  “All new patients recruited after the conclusions of the interim analysis are made, will be randomised in a (2:) 2: 1 ratio to the selected regimen(s) of propranolol or placebo until a total of (100:)100: 50 patients (or more in the case where a sample size increase is recommended) have been randomised over the two stages of the study.”^98^ Extracted from supplementary material. (2:) and (100:) are only applicable if the second best regimen is selected at stage 1.
*Example 2. Bayesian RAR; pre-planned algorithm to update allocation ratios; frequency of updates (after every participant);no burn-in period; period of a fixed control allocation ratio; information that informed adaptation; decision-making criteria for dropping treatments (part of item 7b)*
  See Appendix C as extracted from Giles et al.^71^
*Example 3. Bayesian RAR; burn-in period; fixed control allocation ratio; details of adaptive randomisation including additional adaptations and decision-making criteria (part of item 7b); derivation of statistical quantities; details of Bayesian models and prior distribution with rationale*
  “…eligible patients were randomized on day 1 to treatment with placebo or neublastin 50, 150, 400, 800, or 1200 mg/kg, administered by intravenous injection on days 1, 3, and 5. The first 35 patients were randomized in a 2:1:1:1:1:1 ratio to placebo and each of the 5 active doses (randomisation method required) (i.e., 10 patients in the placebo group and 5 for each dose of active treatment). Subsequently, 2 of every 7 enrolled patients were assigned to placebo. Interim data evaluations of pain (AGPI) and pruritus questionnaire data (proportion of patients who reported ‘the itch is severe enough to cause major problems for me’ on an Itch Impact Questionnaire) were used to update the allocation probability according to a Bayesian algorithm for adaptive allocation and to assess efficacy and futility criteria for early stopping of enrolment (fig. 1 [not shown here]). Interim evaluations and updates to the allocation probabilities were performed weekly. Enrolment was to be stopped early after ≥50 patients had been followed for 4 weeks if either the efficacy criterion (>80% probability that the maximum utility dose reduces the pain score by ≥1.5 points more than the placebo) or the futility criterion (<45% probability that the maximum utility dose reduces pain more than the placebo) was met.”^140^ Details of statistical models used—including computation of posterior quantities; prior distribution with rationale; generation of the utility function; and weighting of randomisation probabilities—are accessible via a weblink provided (https://links.lww.com/PAIN/A433).

### Section 11. Randomisation (Blinding)

*ACE item 11c (new): Measures to safeguard the confidentiality of interim information and minimise potential operational bias during the trial*

*Explanation*—Preventing or minimising bias is central for robust evaluation of the beneficial and harmful effects of interventions. Analysis of accumulating trial data brings challenges regarding how knowledge or leakage of information, or mere speculation about interim treatment effects, may influence behaviour of key stakeholders involved in the conduct of the trial.^22^ ^122^ ^200^ Such behavioural changes may include differential clinical management; reporting of harmful effects; clinical assessment of outcomes; and decision-making to favour one treatment group over the other. Inconsistencies in trial conduct before and after adaptations have wide implications that may affect trial validity and integrity.^22^ For example, use of statistical methods that combine data across stages may become questionable or may make overall results uninterpretable. AD randomised trials whose integrity was severely compromised by disclosure of interim results have resulted in regulators questioning the credibility of conclusions.^201^ ^202^ Most AD randomised trials, 76% (52/68)^49^ and 60% (151/251),^112^ did not disclose methods to minimise potential operational bias during interim analyses. The seriousness of this potential risk will depend on various trial characteristics, and the purpose of having disclosure is to enable readers to judge the risk of potential sources of bias, and thus judge how trustworthy they can assume results to be.

The literature covers processes and procedures which could be considered by researchers to preserve confidentiality of interim results to minimise potential operational bias.^45^ ^123^ ^203^ There is no universal approach that suits every situation due to factors such as feasibility; nature of the trial; and available resources and infrastructure. Some authors discuss roles and activities of independent committees in adaptive decision-making processes and control mechanisms for limiting access to interim information.^203^ ^204^ ^205^

Description of the process and procedures put in place to minimise the potential introduction of operational bias related to interim analyses and decision-making to inform adaptations is essential.^22^ ^125^ ^203^ Specifically, authors should give consideration to:

- Who recommended or made adaptation decisions. The roles of the sponsor or funder, clinical investigators, and trial monitoring committees (for example, independent data monitoring committee or dedicated committee for adaptation) in the decision-making process should be clearly stated;
- Who had access to interim data and performed interim analyses;
- Safeguards which were in place to maintain confidentiality (for example, how the interim results were communicated and to whom and when).

See box 15 for exemplars.

Box 15 **Exemplars on reporting item 11c elements**

*Example 1. Inferentially seamless phase 2/3 AD*
  “The interim analysis was carried out by an independent statistician (from ClinResearch GmbH, Köln, Germany), who was the only person outside the Data Monitoring Committee (DMC) with access to the semi-blinded randomization (sic) codes (treatment groups identified by letters A to G). This statistician functioned independently of the investigators, the sponsor’s clinical trial team members and the team that produced statistical programming for the interim analysis (DATAMAP GmbH, Freiburg, Germany). The independent statistician was responsible for all analyses of efficacy and safety data for the interim analysis. The DMC was given semi-blinded results with treatment groups identified by the letters A to G, with separate decodes sealed in an envelope to be opened for decision-making. The personnel involved in the continuing clinical study were told which two doses had been selected, but study blinding remained in place and the results of the interim analysis were not communicated. No information on the effects of the indacaterol doses (including the two selected) was communicated outside the DMC.”^141^
*Example 2. Bayesian inferentially seamless phase 2/3 AD with RAR*
  “An independent Data Monitoring Committee (DMC) external to Lilly provided oversight of the implementation of the adaptive algorithm and monitored study safety. The DMC fulfilled this role during the dose-finding portion, and continued monitoring after dose selection until an interim database lock at 52 weeks, at which time the study was unblinded to assess the primary objectives. Sites and patients continued to be blinded to the treatment allocation until the completion of the study. The DMC was not allowed to intervene with the design operations. A Lilly Internal Review Committee (IRC), independent of the study team, would meet if the DMC recommended the study to be modified. The role of the IRC was to make the final decision regarding the DMC’s recommendation. The external Statistical Analysis Center (SAC) performed all interim data analyses for the DMC, evaluated the decision rules and provided the randomization updates for the adaptive algorithm. The DMC chair and the lead SAC statistician reviewed these (interim) reports and were tasked to convene an unscheduled DMC meeting if an issue was identified with the algorithm or the decision point was triggered.”^97^
*Example 3. Inferentially seamless phase 2/3 AD with treatment selection, SSR, and non-binding futility stopping*
  “Following the interim analysis of the data and the review of initial study hypotheses, the committee (IDMC) chairman will recommend in writing to the sponsor whether none, one or two regimen(s) of propranolol is (are) considered to be the ‘best’ (the most efficacious out of all regimens with a good safety profile) for further study in stage two of the design. The second ‘best’ regimen will only be chosen for further study along with the ‘best’ regimen if the first stage of the study suggests that recruitment in the second stage will be too compromised by the fact that 1 in 3 patients are assigned to placebo. The IDMC will not reveal the exact sample size increase in the recommendation letter in order to avoid potential sources of bias (only the independent statistician, the randomisation team and the IP suppliers will be informed of the actual sample size increase). Any safety concerns will also be raised in the IDMC recommendation letter. The chairman will ensure that the recommendations do not unnecessarily unblind the study. In the case where the sponsor decides to continue the study, the independent statistician will communicate to the randomisation team which regimen(s) is (are) to be carried forward.”^98^ Extracted from supplementary material.

### Section 12. Statistical methods

*CONSORT 2010 item 12a: Statistical methods used to compare groups for primary and secondary outcomes*
*ACE item 12a (modification): Statistical methods used to compare groups for primary and secondary outcomes, and any other outcomes used to make pre-planned adaptations*

*Comment*—This item should be applied with reference to the detailed discussion in the CONSORT 2010 statement.^3^ ^4^

*Explanation*—The CONSORT 2010 statement^3^ ^4^ addresses the importance of detailing statistical methods to analyse primary and secondary outcomes at the end of the trial. This ACE modified item extends this to require similar description to be made of statistical methods used for interim analyses. Furthermore, statistical methods used to analyse any other adaptation outcomes (item 6) should be detailed to enhance reproducibility of the adaptation process and results. Authors should focus on complete description of statistical models and aspects of the estimand of interest^206^ ^207^ consistent with stated objectives and hypotheses (item 2b) and pre-planned adaptations (item 3b).

For Bayesian ADs, item 12b (paragraph 6) describes similar information that should be reported for Bayesian methods.

See box 16 for exemplars.

Box 16 **Exemplars on reporting item 12a elements**

*Example 1. Frequentist AD*
  Authors are referred to the CONSORT 2010 statement^3^ ^4^ for examples.
*Example 2. 2-stage Bayesian biomarker-based AD with RAR*
  In a methods paper, Gu et al^208^ detail Bayesian logistic regression models for evaluating treatment and marker effects at the end of stage 1 and 2 using non-informative normal priors during RAR and futility early stopping decisions. Strategies for variable selection and model building at the end of stage 1 to identify further important biomarkers for use in RAR of stage 2 patients are described (part of item 3b), including a shrinkage prior used for biomarker selection with rationale.

*ACE item 12b (new): For the implemented adaptive design features, statistical methods used to estimate treatment effects for key endpoints and to make inferences*

*Comments—*Note that items 7a and 12b are connected. Key endpoints are all primary endpoints as well as other endpoints considered highly important, for example, an endpoint used for adaptation.

*Explanation*—A goal of every trial is to provide reliable estimates of the treatment effect for assessing benefits and risks to reach correct conclusions. Several statistical issues may arise when using an AD depending on its type and the scope of adaptations, the adaptive decision-making criteria and whether frequentist or Bayesian methods are used to design and analyse the trial.^22^ Conventional estimates of treatment effect based on fixed design methods may be unreliable when applied to ADs (for example, may exaggerate the patient benefit).^96^ ^209^ ^210^ ^211^ ^212^ ^213^ Precision around the estimated treatment effects may be incorrect (for example, the width of confidence intervals may be incorrect). Other methods available to summarise the level of evidence in hypothesis testing (for example, p-values) may give different answers. Some factors and conditions that influence the magnitude of estimation bias have been investigated and there are circumstances when it may not be of concern.^209^ ^214^ ^215^ ^216^ ^217^ ^218^ Secondary analyses (for example, health economic evaluation) may also be affected if appropriate adjustments are not made.^219^ ^220^ Cameron et al^221^ discuss methodological challenges in performing network meta-analysis when combining evidence from randomised trials with ADs and fixed designs. Statistical methods for estimating the treatment effect and its precision exist for some ADs^68^ ^222^ ^223^ ^224^ ^225^ ^226^ ^227^ ^228^ ^229^ ^230^ ^231^ and implementation tools are being developed.^82^ ^232^ ^233^ ^234^ However, these methods are rarely used or reported and the implications are unclear.^49^ ^209^ ^235^ Debate and research on inference for some ADs with complex adaptations is ongoing.

In addition to statistical methods for comparing outcomes between groups (item 12a), we specifically encourage authors to clearly describe statistical methods used to estimate measures of treatment effects with associated uncertainty (for example, confidence or credible intervals) and p-value (when appropriate); referencing relevant literature is sufficient. When conventional or naïve estimators derived from fixed design methods are used, it should be clearly stated. In situations where statistical simulations were used to either explore the extent of bias in estimation of the treatment effects (such as^181^ ^236^) or operating characteristics, it is good practice to mention this and provide supporting evidence (item 24c).

ADs tend to increase the risk of making misleading or unjustified claims of treatments effects if traditional methods that ignore trial adaptations are used. In general, this arises when selecting one or more hypothesis test results from a possible list in order to claim evidence of the desired conclusion. For instance, the risks may increase by testing the same hypothesis several times (for example, at interim and final analyses), hypothesis testing of multiple treatment comparisons, selecting an appropriate population from multiple target populations, adapting key outcomes, or a combination of these.^22^ A variety of adaptive statistical methods exist for controlling specific operating characteristics of the design (for example, type I error rate, power) depending on the nature of the repeated testing of hypotheses.^51^ ^61^ ^62^ ^82^ ^192^ ^237^ ^238^ ^239^ ^240^ ^241^ ^242^

Authors should therefore state operating characteristics of the design that have been controlled and details of statistical methods used. The need for controlling a specific type of operating characteristic (for example, pairwise or familywise type I error rate) is context dependent (for example, based on regulatory considerations, objectives and setting) so clarification is encouraged to help interpretation. How evidence of benefit and/or risk is claimed (part of item 7a) and hypotheses being tested (item 2b) should be clear. In situations where statistical simulations were used, we encourage authors to provide a report, where possible (item 24b).

When data or statistical tests across independent stages are combined to make statistical inference, authors should clearly describe the combination test method (for example, Fisher’s combination method, inverse normal method or conditional error function)^192^ ^240^ ^241^ ^243^ ^244^ and weights used for each stage (when not obvious). This information is important because different methods and weights may produce results that lead to different conclusions. Bauer and Einfalt^107^ found low reporting quality of these methods.

Brard et al^245^ found evidence of poor reporting of Bayesian methods. To address this, when a Bayesian AD is used, authors should detail the model used for analysis to estimate the posterior probability distribution; the prior distribution used and rationale for its choice; whether the prior was updated in light of interim data and how; and clarify the stages when the prior information was used (interim or/and final analysis). If an informative prior was used, the source of data to inform this prior should be disclosed where applicable. Of note, part of the Bayesian community argue that it is not principled to control frequentist operating characteristics in Bayesian ADs,^246^ although these can be computed and presented.^22^ ^154^ ^247^

Typically, ADs require quickly observed adaptation outcomes relative to the expected length of the trial. In some ADs, randomised participants who have received the treatment may not have their outcome data available at the interim analysis (referred to as overrunning participants) for various reasons.^248^ These delayed responses may pose ethical dilemmas depending on the adaptive decisions taken, present logistical challenges, or diminish the efficiency of the AD depending on their prevalence and the objective of the adaptations.^201^ It is therefore useful for readers to understand how overrunning participants were dealt with at interim analyses especially after a terminal adaptation decision (for example, when a trial or treatment groups were stopped early for efficacy or futility). If outcome data of overrunning participants were collected, a description should be given of how these data were analysed and combined with interim results after the last interim decision was made. Some formal statistical methods to deal with accrued data from overrunning participants have been proposed.^249^

See box 17 for exemplars.

Box 17 **Exemplars on reporting item 12 elements**

*Example 1. GSD; statistical method for estimating treatment effects*
  “Stagewise ordering was used to compute the unbiased median estimate and confidence limits for the prognosis-group-adjusted hazard rates.^250^”^251^
*Example 2. Inferentially seamless (4-arm 2-stage) AD with dose selection; statistical methods for controlling operating characteristics*
  “…the power of the study ranged from 71% to >91% to detect a treatment difference at a one-sided α of 0.025 when the underlying response rate of ≥1 of the crofelemer dose groups exceeded placebo by 20%. The clinical response of 20% was based on an estimated response rate of 55% in crofelemer and 35% in placebo during the 4-week placebo-controlled assessment period.… For the primary endpoint, the test for comparing the placebo and treatment arms reflected the fact that data were gathered in an adaptive fashion and controlled for the possibility of an increased Type I error rate. Using the methods of Posch and Bauer,^68^ as agreed upon during the special protocol assessment process, a p-value was obtained for comparison of each dose to the placebo arm from the stage I data, and an additional p-value was obtained for comparison of the optimal dose to the placebo arm from the independent data gathered in stage II. For the final primary analysis, the p-values from the first and second stages were combined by the inverse normal weighting combination function, and a closed testing procedure was implemented to test the null hypothesis using the methods of Posch and Bauer,^68^ based on the original work of Bauer and Kieser.^69^ This closed test controlled the experiment-wise error rate for this 2-stage adaptive design at a one-sided α of 0.025.”^252^ Extracted from appendix material.
*Example 3. 3-arm 2-stage group-sequential AD with treatment selection; combination test method; multiplicity adjustments; statistical method for estimating treatment effects*
  “The proposed closed testing procedure will combine weighted inverse normal combination tests using pre-defined fixed weights, the closed testing principle,^68^ ^253^ ^254^ and the Hochberg-adjusted 1-sided P-value on stage 1 data. This testing procedure strongly controls the overall type I error rate at α level (see “Simulations run to assess the type I error rate under several null hypothesis scenarios”). Multiplicity-adjusted flexible repeated 95% 2-sided CIs^217^ on the percentage of patients will be calculated for otamixaban dose 1, otamixaban dose 2, and UFH plus eptifibatide. Relative risk and its 95% 2-sided CIs will also be calculated. Point estimates based on the multiplicity-adjusted flexible repeated CIs will be used.”^181^ See supplementary material of the paper for details.
*Example 4. Population-enrichment AD with SSR; criteria for claiming evidence of benefit; methods for controlling familywise type I error; combination test weights*
  Mehta et al^99^ published a methodological paper detailing a family of three hypotheses being tested; use of closure testing principle^254^ to control the overall type I error; how evidence is claimed; and analytical derivations of the Simes adjusted p-values.^255^ This includes the use of a combination test approach using pre-defined weights based on the accrued information fraction for the full population (cutaneous and non-cutaneous patients) and subpopulation (cutaneous patients). Analytical derivations were presented for the two cases assuming enrichment occurs at interim analysis and no enrichment after interim analysis. Details are reported in a supplementary file accessible via the journal website.
*Example 5. Inferentially seamless (7-arm 2-stage) AD with dose selection; use of traditional naïve estimates*
  “Unless otherwise stated, efficacy data are given as least squares means with standard error (SE) or 95% confidence interval (CI).”^80^
*Example 6. Inferentially seamless phase 2/3 (5-arm 2-stage) AD with dose selection; dealing with overrunning participants*
  “Patients already assigned to an unselected regimen of propranolol by the time that the conclusions of the interim analysis are available, will continue the treatment according to the protocol but efficacy data for these patients will not be included in the primary analysis of primary endpoint.”^98^ Extracted from the supplementary material.

### Section 13. Results (Participant flow)

*CONSORT 2010 item 13a: For each group, the numbers of participants who were randomly assigned, received intended treatment, and were analysed for the primary outcome*
*ACE item 13a (modification): For each group, the numbers of participants who were randomly assigned, received intended treatment, and were analysed for the primary outcome and any other outcomes used to inform pre-planned adaptations, if applicable*

*Comments*—Authors are referred to the CONSORT 2010 statement^3^ ^4^ for detailed discussion. Here, we only address additional requirements for ADs.

*Explanation*—The CONSORT 2010 statement^3^ ^4^ discusses why it is essential to describe participant flow adequately from screening to analysis. This applies to both interim and final analyses depending on the stage of reporting. The number of participants for each group with adaptation outcome data (that contributed to the interim analyses) should also be reported if different from the number of participants with primary outcome data. Furthermore, authors should report the number of randomised participants, for each group, that did not contribute to each interim analysis because of lack of mature outcome data at that interim look. For example, overrunning participants that were still being followed up when a terminal adaptation decision was made (for example, dropping of treatment groups or early trial termination). The presentation of participant flow should align with the key hypotheses (for example, subpopulation(s) and full study population) and treatment comparisons depending on the stage of results being reported.

See box 18 for exemplars.

Box 18 **Exemplars on reporting item 13 (participant flowcharts)**

*Example 1. Inferentially seamless phase 2/3 AD*
  Appendix D is an illustrative structure that could be used to show the flow of participants when reporting the final results from a trial such as ADVENT.^252^
*Example 2. Population enrichment AD*
  Appendices E and F illustrate participant flowcharts that could be used for a population-enrichment adaptive trial such as TAPPAS,^99^ which had key hypotheses relating to the cutaneous subpopulation and full population (cutaneous and non-cutaneous) depending on whether enrichment was done or not.
*Example 3. Bayesian biomarker-targeted AD with RAR*
  Appendix G is an adapted flow diagram from BATTLE^256^ showing the number of participants that contributed to the analysis by biomarker group (subpopulations) during fixed randomisation (burn-in period) followed by RAR.
*Example 4. MAMS AD*
  Appendix H can be adapted for reporting a MAMS trial such as TAILoR.^257^

### Section 14. Results (Recruitment)

*CONSORT 2010 item 14a: Dates defining the periods of recruitment and follow-up*
*ACE item 14a (modification): Dates defining the periods of recruitment and follow-up, for each group*

*Comment*—Authors should refer to the CONSORT 2010 statement^3^ ^4^ for the discussion.

*Explanation*—Consumers of research findings should be able to put trial results, study interventions, and comparators into context. Some ADs, such as those that evaluate multiple treatments allowing dropping of futile ones, selection of promising treatments, or addition of new treatments to an ongoing trial,^19^ ^106^ ^258^ ^259^ incorporate pre-planned adaptations to drop or add new treatment groups during the course of the trial. As a result, dates of recruitment and follow-up may differ across treatment groups. In addition, the comparator arm may also change with time and concurrent or non-concurrent controls may be used. There are statistical implications that include how analysis populations for particular treatment comparisons are defined at different stages. For each treatment group, authors should clearly state the exact dates defining recruitment and follow-up periods. It should be stated if all treatment groups were recruited and followed-up during the same period.

See box 19 for exemplars.

Box 19 **Exemplars on reporting item 14a**

**Fig 2:**
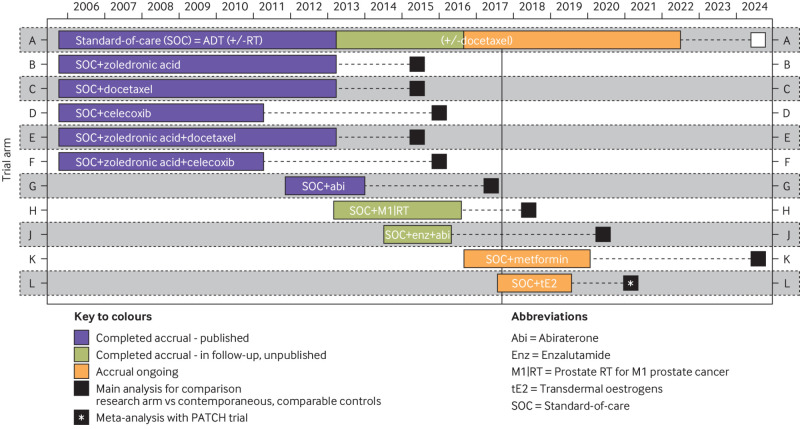
Redrawn from Gilson et al.^260^ Reused in accordance with the terms of Creative Commons Attribution 4.0 International License (https://creativecommons.org/licenses/by/4.0/). No changes to the original figure were made.

*Example 1. MAMS platform AD*
Figure 2 illustrates the graphical reporting of recruitment and follow-up periods for each treatment group including new arms that were added during the STAMPEDE trial. Corresponding comparator groups (controls) for treatment comparisons are indicated.

*Example 2. Phase 2 Bayesian biomarker-targeted AD with RAR*
  “A total of 341 patients were enrolled in the BATTLE study between November 30, 2006, and October 28, 2009, with equally random assignments for the first 97 patients and adaptive randomization for the remaining 158.”^256^

*CONSORT 2010/ACE item 14b (clarification): Why the trial ended or was stopped*

*Comment*—This item should be applied without reference to the CONSORT 2010 statement.^3^ ^4^

*Explanation*—Some clinical trials are stopped earlier than planned for reasons that will have implications for interpretation and generalisability of results. For example, poor recruitment is a common challenge.^261^ This may limit the inference drawn or complicate interpretation of results based on insufficient or truncated trial data. Thus, the reporting of reasons for stopping a trial early including circumstances leading to that decision could help readers to interpret results with relevant caveats.

The CONSORT 2010 statement,^3^ ^4^ however, did not distinguish early stopping of a trial due to a pre-planned adaptation from an unplanned change. To address this and for consistency, we have now reserved this item for reporting of reasons why the trial or certain treatment arm(s) were stopped outside the scope of pre-planned adaptations, including those involved in deliberations leading to this decision (for example, sponsor, funder, or trial monitoring committee). We also introduced item 14c to capture aspects of adaptation decisions made in light of the accumulating data, such as stopping the trial or treatment arm because the decision-making criterion to do so has been met.

See box 20 for exemplars.

Box 20 **Exemplars on reporting item 14b**

*Example 1. 2-stage AD with options for futility and efficacy early stopping and increase in sample size; unplanned trial termination*
  “The planned interim analysis of the study was done in November 2005 after 125 patients have been (sic) recruited.… According to the adaptive design of the study, we therefore calculated another 163 patients per treatment group to be required to answer the primary question. Upon the slow accrual up to that timepoint, the study coordinators decided to close the trial at the end of 2005. Further analysis was regarded to be exploratory.”^193^
*Example 2. Sequential-step AD; unplanned trial termination*
  “…the third interim analysis indicated unexpectedly low initial cure rates in both arms; 84% in the multiple dose and 73% in the single-dose arm. The stopping rule was not met …, but based on the observed poor efficacy overall, and following discussions with the Data Safety and Monitoring Board (DSMB) and investigators, the sponsor terminated the trial.”^262^

*ACE item 14c (new): Specify what trial adaptation decisions were made in light of the pre-planned decision-making criteria and observed accrued data*

*Explanation*—ADs depend on adherence to pre-planned decision rules to inform adaptations. Thus, it is vital for research consumers to be able to assess whether the adaptation rules were adhered to as pre-specified in the decision-making criteria given the observed accrued data at the interim analyses. Failure to adhere to pre-planned decision rules may undermine the integrity of the results and validity of the design by affecting the operating characteristics (see item 7b for details on binding and non-binding decision rules).

Unforeseeable events can occur that may lead to deviations from some pre-planned adaptation decisions rules (for example, the overruling or ignoring of certain rules). It is therefore essential to adequately describe which pre-planned adaptations were enforced, which were pre-planned but were not enforced or overruled even though the interim analysis decision rules indicated an adaptation should be made, and which unplanned changes were made other than unplanned early stopping of the trial or treatment arm(s) covered by item 14b. Pre-planned adaptations that were not implemented are difficult to assess because the interim decisions made versus the pre-planned intended decisions are often poorly reported, and reasons are rarely given.^115^ The rationale for ignoring or overruling pre-planned adaptation decisions, or making unplanned decisions that affect the adaptations should be clearly stated and also who recommended or made such decisions (for example, the data monitoring committee or adaptation committee). This enables assessment of potential bias in the adaptation decision-making process, which is crucial for the credibility of the trial.

Authors should indicate the point at which the adaptation decisions were made (that is, stage of results) and any additional design changes that were made as a consequence of adaptation decisions (for example, change in allocation ratio).

See box 21 for exemplars.

Box 21 **Exemplars on reporting item 14c elements**

*Example 1. Bayesian adaptive-enrichment AD with futility and superiority early stopping; stage of results*
  “Enrolment in the trial was stopped at 31 months, because the results of an interim analysis met the pre-specified criterion for trial discontinuation, which was a predictive probability of superiority of thrombectomy of at least 95% for the first primary endpoint (the mean score for disability on the utility-weighted modified Rankin scale at 90 days). This was the first pre-specified interim analysis that permitted stopping for this reason, and it was based on the enrolment of 200 patients. Because enrichment thresholds had not been crossed, the analysis included the full population of patients enrolled in the trial, regardless of infarct volume.”^100^
*Example 2. Dose-selection decisions for an inferentially seamless phase 2/3 AD*
  “The two doses of indacaterol selected against the two reference efficacy criteria were 150 µg (as the lowest dose exceeding both criteria) and 300 µg (as the next highest dose). The safety results, together with the safety data from the other 1-year study, led the DMC to conclude that there was no safety signal associated with indacaterol at any dose. Thus, the two doses selected (at stage 1) to continue into stage 2 of the study were indacaterol 150 and 300 µg.”^141^

### Section 15. Results (Baseline data)

*CONSORT 2010 item 15: A table showing baseline demographic and clinical characteristics for each group*
*ACE Item 15a «15 (clarification, renumbered): A table showing baseline demographic and clinical characteristics for each group*

*Comments*—We renumbered the item to accommodate the new item 15b. This item should be applied with reference to the CONSORT 2010 statement,^3^ ^4^ with additional requirements for specific ADs.

*Explanation*—The presentation of treatment group summaries of key characteristics and demographics of randomised participants who contributed to results influences interpretation and helps readers and medical practitioners to make judgements about which patients the results are applicable to. For some ADs, such as population (or biomarker or patient) enrichment,^87^ ^146^ when the study population is considered heterogeneous, a trial could be designed to evaluate if study treatments are effective in specific pre-specified subpopulations or a wider study population (full population). A pre-planned adaptation strategy may involve testing the effect of treatments in both pre-specified subpopulations of interest and the wider population in order to target patients likely to benefit the most. For such ADs, it is essential to provide summaries of characteristics of those who were randomised and who contributed to the results being reported (both interim or final), by treatment group for each subpopulation of interest and the full population consistent with hypotheses tested. These summaries should be reported without hypothesis testing of baseline differences in participants’ characteristics because it is illogical in randomised trials.^263^ ^264^ ^265^ ^266^ The CONSORT 2010 statement^3^ ^4^ presents an example of how to summarise baseline characteristics.

In the presence of marked differences in the numbers of randomised participants and those included in the interim or final analyses, authors are encouraged to report baseline summaries by treatment group for these two populations. Readers will then be able to assess representativeness of the interim or final analysis population relative to those randomised and also the target population.

See box 22 for an exemplar.

Box 22 **Exemplar on reporting item 15a**

*Example. Population-enrichment AD*
  See Appendix I for a dummy baseline table for the TAPPAS trial.^99^

*ACE item 15b (new): Summary of data to enable the assessment of similarity in the trial population between interim stages*

*Comment*—This item is applicable for ADs conducted in distinct stages for which the trial has progressed beyond the first stage.

*Explanation*—Changes in trial conduct and other factors may introduce heterogeneity in the characteristics or standard management of patients before and after trial adaptations. Consequently, results may be inconsistent or heterogeneous between stages (interim parts) of the trial.^201^ For ADs, access to interim results or mere guesses based on interim decisions taken may influence behaviour of those directly involved in the conduct of the trial and thus introduce operational bias.^22^ Some trial adaptations may introduce intended changes to inclusion or exclusion criteria (for example, population enrichment^92^ ^146^). Unintended changes to characteristics of patients over time may occur (population drift).^267^ A concern is whether this could lead to a trial with a different study population that does not address the primary research objectives.^268^ This jeopardises validity, interpretability, and credibility of trial results. It may be difficult to determine whether differences in characteristics between stages occurred naturally due to chance, were an unintended consequence of pre-planned trial adaptations, represent operational bias introduced by knowledge or communication of interim results, or are for other reasons.^269^ However, details related to item 11c may help readers make informed judgements on whether any observed marked differences in characteristics between stages are potentially due to systematic bias or just chance. Therefore, it is essential to provide key summary data of participants included in the analysis (as discussed in item 15a) for each interim stage of the trial and overall. Authors are also encouraged to give summaries by stage and treatment group. This will help readers assess similarity in the trial population between stages and whether it is consistent across treatment groups.

See box 23 for an exemplar.

Box 23 **Exemplar on reporting item 15b elements**

*Example. Overall baseline characteristics by stage; inferentially seamless phase 2/3 AD*. See table 5

**Table 5:** Characteristics of randomised participants (N=1202) in stage 1 and 2.

Characteristic	Stage 1 (n=230)	Stage 2 (n=972)
Age (years), mean (SD)	53.4 (10.3)	54.3 (9.7)
Gender (female), n (%)	139 (60.4)	504 (51.9)
Race (white), n (%)	103 (44.8)	509 (52.4)
BMI (kg/m^2^), mean (SD)	31.9 (4.5)	31.1 (4.3)
Body weight (kg), mean (SD)	87.3 (18.0)	86.2 (17.1)
Duration of diabetes (years), mean (SD)	7.5 (5.5)	7.0 (5.1)
Seated systolic BP (mm Hg), mean (SD)	128.0 (14.4)	127.7 (13.1)
Seated diastolic BP (mm Hg), mean (SD)	77.9 (7.9)	77.6 (8.6)
Seated heart rate (bpm), mean (SD)	74.5 (9.6)	75.2 (10.0)

Adapted from Geiger et al^166^; BMI = Body Mass Index; SD = standard deviation; BP = blood pressure; bpm = beats per minute; mm Hg = millimetres of mercury. Data presented were from an ongoing trial so are incomplete and only used for illustration.

### Section 16. Results (Numbers analysed)

*CONSORT 2010/ACE item 16 (clarification): For each group, number of participants (denominator) included in each analysis and whether the analysis was by original assigned groups*

*Comments*—The item should be used in reference to the CONSORT 2010 statement^3^ ^4^ for original details and examples. Here, we give additional clarification for some specific requirements of certain ADs such as population enrichment.^87^ ^146^

*Explanation*—We clarify that the number of participants by treatment group should be reported for each analysis at both the interim analyses and final analysis whenever a comparative assessment is performed (for example, for efficacy, effectiveness, or safety). Most importantly, the presentation should reflect the key hypotheses considered to address the research questions. For example, population (or patient or biomarker) enrichment ADs can be reported by treatment group for each pre-specified subpopulation and full population depending on key hypotheses tested.

### Section 17. Results (Outcomes and estimation)

*CONSORT 2010/ACE item 17a (clarification): For each primary and secondary outcome, results for each group, and the estimated effect size and its precision (such as 95% confidence interval)*

*Comments*—We expanded the explanatory text to address some specific requirements of certain ADs such as population enrichment.^146^ Therefore, the item should be used in reference to the CONSORT 2010^3^ for original details and examples.

*Explanation—*In randomised trials, we analyse participant outcome data collected after study treatments are administered to address research questions about beneficial and/or harmful effects of these treatments. In principle, reported results should be in line with the pre-specified estimand(s) and compatible with the research questions or objectives.^206^ ^207^ The CONSORT 2010 statement^3^ ^4^ addresses what authors should report depending on the outcome measures. These include group summary measures of effect, for both interim and final analyses, including the number of participants contributing to the analysis, appropriate measures of the treatment effects (for example, between group effects for a parallel group randomised trial) and associated uncertainty (such as credible or confidence intervals). Importantly, the presentation is influenced by how the key hypotheses are configured to address the research questions. For some ADs, such as population (or biomarker or patient) enrichment, key hypotheses often relate to whether the study treatments are effective in the whole target population of interest or in specific subpopulations of the target population classified by certain characteristics. In such ADs, reporting of results as detailed in the CONSORT 2010 should mirror hypotheses of interest. That is, we expect the outcome results to be presented for the subpopulations and full target population considered by treatment group. This is to help readers interpret results on whether the study treatments are beneficial to the target population as a whole or only to specific pre-specified subpopulations.

*ACE item 17c (new): Report interim results used to inform interim decision-making*

*Explanation*—Adherence to pre-planned adaptations and decision rules including timing and frequency is essential in AD randomised trials. This can only be assessed when the pre-planned adaptations (item 3b), adaptive decision rules (item 7b), and results that are used to guide the trial adaptations are transparently and adequately reported.

Marked differences in treatment effects between stages may arise (for example, discussed in item 15b) making overall interpretation of their results difficult.^92^ ^110^ ^267^ ^269^ ^270^ ^271^ ^272^ The presence of heterogeneity questions the rationale for combining results from independent stages to produce overall evidence, as is also the case for combining individual studies in a meta-analysis.^92^ ^273^ Although this problem is not unique to AD randomised trials, consequences of trial adaptation may worsen the problem.^269^ Authors should at least report the relevant interim or stage results that were used to make each adaptation, consistent with items 3b and 7b; for example, interim treatment effects with uncertainty, interim conditional power or variability used for SSR, and trend in the probabilities of allocating participants to a particular treatment group as the trial progresses. Authors should report interim results of treatment groups or subpopulations that have been dropped due to lack of benefit or poor safety. This reduces the reporting bias caused by selective disclosure of treatments only showing beneficial and/or less harmful effects.

See box 24 for exemplars.

Box 24 **Exemplars on reporting item 17 elements**

**Fig 3:**
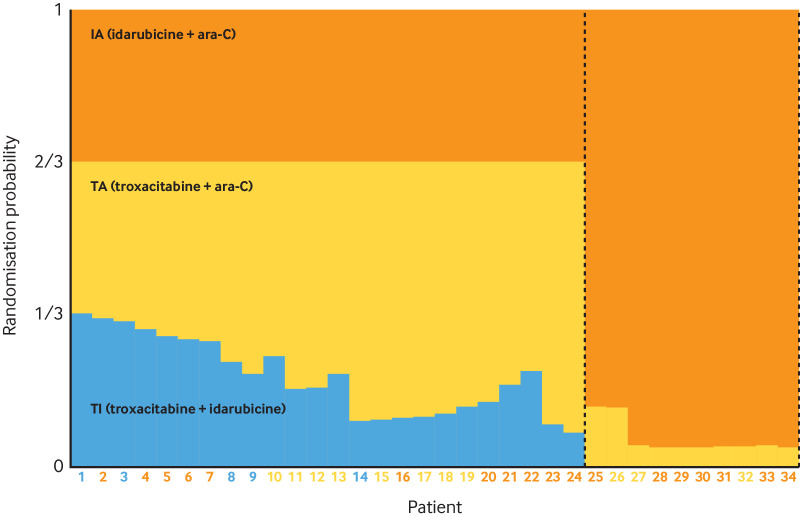
Redrawn from Pallmann et al.^22^ Reused in accordance with the terms of Creative Commons Attribution 4.0 International License (https://creativecommons.org/licenses/by/4.0/). No changes to the original figure were made.

*Example 1. Bayesian RAR; change in randomisation probabilities across arms throughout the trial; randomisation updates were made after every patient*
  Giles et al^71^ present a table of changes in allocation probabilities used to create figure 3 by treatment group including allocated treatment and primary outcome response for each participant.

*Example 2. Inferentially seamless phase 2/3 AD; stage 1 treatment selection results*
  Barnes et al^141^ clearly presented the results that led to the interim selection of the two indacaterol drug doses to progress to stage 2 of the study; 150 µg (the lowest dose that exceeded both pre-specified treatment selection criteria) and 300 µg (the next highest dose that met the same criteria). The interim difference in treatment effect compared to placebo with uncertainty per group for the two adaptation outcomes are displayed in figures 1 and 2 of the paper.
*Example 3. 2-stage GSD; stage 1 dose selection results*
  “At the interim analysis planned after at least 1969 patients had been randomized and reached day 7 follow-up in each group,^181^ the otamixaban dose for stage 2 of the trial was selected as described in eFigure 1 in the Supplement. At that time, the rates of the primary efficacy outcome in the higher-dose otamixaban group was xx (4.7%) (the one selected to go forward) and was xx (5.6%) in the UFH-pluseptifibatide group (adjusted RR, 0.848; 95% CI, 0.662-1.087) but the lower-dose group fulfilled the pre-specified criteria for futility with a RR of more than 1 (primary efficacy outcome, xx (6.3%); RR, 1.130; 95% CI, 0.906-1.408) and was discontinued.”^144^ xx are the corresponding number of participants with primary response that should have been stated.
*Example 4. Adapted from Khalil et al* 143 *; sequential-step AD*. See table 6.

**Table 6:** Interim results

Parasite clearance at day 30 (initial cure)	Treatment group	Parasite clearance rate, n/N (%)	Differences in parasite clearance rates (95% CI)	P-value
Interim analysis 1	Single dose, 7.5mg/kg	10/20 (50.0%)	Reference	
	Multiple dose, 7x3mg/kg	16/18 (88.9%)	38.9% (12.6 to 65.2)	0.015^a^
Interim analysis 2^b^	Single dose, 10mg/kg	16/20 (80.0%)	Reference	
	Multiple dose, 7x3mg/kg	19/25 (76.0%)	−4.0% (−28.2 to 20.2)	0.748^c^
Interim analysis 3^d^	Single dose, 10mg/kg	29/40 (72.5%)	Reference	
	Multiple dose, 7x3mg/kg	37/44 (84.1%)	11.6% (−6.0 to 29.1)	0.196^e^

N, total number of patients per group (denominator); n, patients with recorded parasitic clearance per groups (events); CI, confidence interval; ^a^ p-value from Fisher’s exact test, adaptation rule met to escalate dose so dosage increased to 10 mg/kg and continue recruitment; ^b^ adaptation rule to escalate dose not met so recruitment was continued with the same dosage (10mg/kg in single-dose arm; ^c, e^ p-values from a Chi-square test; ^e^ adaptation rule to escalate dose not met but concerns arose regarding low cure in each arm and recruitment was terminated; ^d^ includes patients in interim analysis 2; patients in interim analysis 1 did not contribute to any subsequent interim analysis.

### Section 20. Discussion (Limitations)

*CONSORT 2010/ACE item 20 (clarification): Trial limitations, addressing sources of potential bias, imprecision, and, if relevant, multiplicity of analyses*

*Comments*—No change in wording is made to this item so it should be applied with reference to the CONSORT 2010 statement^3^ ^4^ for original details and examples. Here, we only address additional considerations for ADs.

*Explanation—*We expect authors to discuss the arguments for and against the implemented study design and its findings. Several journals have guidelines for structuring the discussion to prompt authors to discuss key limitations with possible explanations. The CONSORT 2010 statement^3^ ^4^ addresses general aspects relating to potential sources of bias, imprecision, multiplicity of analyses and implications of unplanned changes to methods or design. For AD randomised trials, further discussion should include the implications of:

- Any deviations from the pre-planned adaptations (for example, decision rules that were not enforced or overruled and changes in timing or frequency of interim analyses);
- Interim analyses (for example, updating randomisation with inadequate burn-in period);
- Protocol amendments on the trial adaptations and results;
- Potential sources of bias introduced by interim analyses or decision-making;
- Potential bias and imprecision of the treatment effects if naïve estimation methods were used;
- Potential heterogeneity in patient characteristics and treatment effects between stages;
- Whether outcome data (for example, efficacy and safety data) were sufficient to robustly inform trial adaptations at interim analyses and;
- Using adaptation outcome(s) different from the primary outcome(s).

Additionally, it is encouraged to discuss the observed efficiencies of pre-planned adaptations in addressing the research questions and lessons learned about using the AD, both negative and positive. This is optional as it does not directly influence the interpretation of the results but enhances much-needed knowledge transfer of innovative trial designs. Therefore, authors have been encouraged to consider separate methodology publications in addition to trial results.^58^ ^181^

See box 25 for exemplars.

Box 25 **Exemplars on reporting item 20**

*Example 1. Use of surrogate outcome to inform adaptation*
  “We chose change in the SOFA scores as a surrogate outcome based on strong correlations between this measure and 28-day mortality (33). Whether change in the SOFA scores and the timing of reassessment (48 hours in this case) represents the “right” surrogate endpoint for nonpivotal sepsis trials remains unclear and is an area for future consideration, although the use of change in the SOFA score as a surrogate outcome is supported by a recent meta-analysis (34).”^142^
*Example 2. Duration of assessments to inform dose selection*
  “The use of the adaptive seamless design is not without potential risk. The initial dose-finding period needs to be long enough for a thorough evaluation of effects. Two weeks was considered a fully adequate period in which to attain pharmacodynamic steady state….”^141^
*Example 3. Early stopping outside the scope of the pre-planned adaptation and possible explanation*
  “The aim was to determine the minimum efficacious dose and safety of treatments in HIV-uninfected patients. However, the study had to be prematurely terminated due to unacceptably low efficacy in both the single and multiple dose treatment arms, with a cure rate of only 85% in the multiple-dose arm. Adverse effects of treatment in this study were in line with the current drug label. The overall low efficacy was unexpected, as total doses of 10 mg/kg and above resulted in DC rates of at least 90% in a trial in Kenya (13). The trial was not powered for data analysis by geographical location (centre) and the results may have been due to chance, but both the 10 mg/kg single dose and 21 mg/kg multiple dose regimens appeared to work very well in the small number of patients treated in Arba Minch Hospital (southern Ethiopia). We have little explanation for the overall poor response seen in this study or for the observed geographical variations. Previously, similar geographical variation in treatment response in these three sites was seen for daily doses of 11 mg/kg body weight paromomycin base over 21 days (7), a regimen which had also proven efficacious in India (18). Methodological bias is unlikely in this randomized trial, but differences in base line patient characteristics between the three trial sites could have possibly introduced bias, leading to variation in treatment response….”^143^
*Example 4. Limitations of biomarkers and RAR*
  “Our study has some important limitations. First, and probably most important, our biomarker groups were less predictive than were individual biomarkers, which diluted the impact of strong predictors in determining treatment probabilities. For example, EGFR mutations were far more predictive than was the overall EGFR marker group. The unfortunate decision to group the EGFR markers also impacted the other marker groups and their interactions with other treatments, resulting in a suboptimal overall disease control rate as described. Second, several of the pre-specified markers (for example, RXR) had little, if any, predictive value in optimizing treatment selections. This limitation will be addressed in future studies by not grouping or prespecifying biomarkers prior to initiating these biopsy-mandated trials. In addition, adaptive randomization, which assigns more patients to the more effective treatments within each biomarker group, only works well with a large differential efficacy among the treatments (as evident in the KRAS/BRAF group), but its role is limited without such a difference (for example, in the other marker groups). Allowing prior use of erlotinib was another limitation and biased treatment assignments; in fact, the percentage of patients previously treated with erlotinib steadily increased during trial enrollment. Overall, 45% of our patients were excluded from the 2 erlotinib-containing arms because of prior EGFR TKI treatment. As erlotinib is a standard of care therapy in NSCLC second-line, maintenance, and front-line settings, the number of patients receiving this targeted agent will likely continue to increase.”^256^

### Section 21. Discussion (Generalisability)

*CONSORT 2010/ACE item 21 (clarification): Generalisability (external validity, applicability) of the trial findings*

*Comments*—We have not changed the wording of this item so it should be considered in conjunction with the CONSORT 2010 statement.^3^ ^4^ However, there are additional considerations that may influence the generalisability of results from AD randomised trials.

*Explanation*—Regardless of the trial design, authors should discuss how the results are generalisable to other settings or situations (external validity) and how the design and conduct of the trial minimised or mitigated potential sources of bias (internal validity).^3^ For ADs, there are many factors that may undermine both internal (see item 20 clarifications) and external validity. Trial adaptations are planned with a clear rationale to achieve research goals or objectives. Thus, the applicability of the results may be intentionally relevant to the target population enrolled or pre-specified subpopulation(s) with certain characteristics (subsets of the target population). Specifically, the implemented adaptations and other factors may cause unintended population drift or inconsistencies in the conduct of the trial. Authors should discuss the population to whom the results are applicable including any threats to internal and external validity which are trial dependent based on the implemented adaptations.

See box 26 for exemplars.

Box 26 **Exemplar on reporting item 21 elements**

*Example 1. Bayesian population-enrichment AD with RAR; to whom the results are applicable (full population)*
  “The DAWN trial showed that, among patients with stroke due to occlusion of the intracranial internal carotid artery or proximal middle cerebral artery who had last been known to be well 6 to 24 hours earlier and who had a mismatch between the severity of the clinical deficit and the infarct volume, outcomes for disability and functional independence at 90 days were better with thrombectomy plus standard medical care than with standard medical care alone.”^100^
*Example 2. Phase 2 Bayesian biomarker-targeted AD with RAR; to whom the results are applicable (biomarker specific)*
  “Sorafenib was active against tumors with mutated or wild-type KRAS, but had a worse disease control rate (compared with other study agents) in patients with EGFR mutations. As expected (5–7, 15–17), erlotinib was beneficial in patients with mutated-EGFR tumors. Erlotinib plus bexarotene improved disease control in patients with a higher expression of Cyclin D1, suggesting a potential role for bexarotene in lung cancer treatment (11); similar to sorafenib, the combination also improved disease control in the KRAS-mutant patient population. Future randomized, controlled studies are needed to further confirm the predictive value of these biomarkers.”^256^ Liu and Lee^85^ published details of the design and conduct of this trial.

### Section 24. Other information (Statistical analysis plan and other relevant trial documents)

*ACE item 24b (new): Where the full statistical analysis plan and other relevant trial documents can be accessed*

*Explanation*—Pre-specifying details of statistical methods and their execution including documentation of amendments and when they occurred is good scientific practice that enhances trial credibility and reproducibility of methods, results and inference. The SAP is the principal technical document that details the statistical methods for the design of the study; analysis of the outcomes; aspects that influence the analysis approaches; and presentation of results consistent with the research questions/objectives and estimands^206^ ^207^ in line with the trial protocol (now item 24a). General guidance on statistical principles for clinical trials to consider with the aim to standardise research practice exists.^274^ ^275^ ^276^ AD trials tend to bring additional statistical complexities and considerations during the design and analyses depending on the trial adaptations considered. Access to the full SAP with amendments (if applicable) addressing interim and final analyses is essential. This can be achieved through the use of several platforms such as online supplementary material, online repositories, or referencing published material. This enables readers to access additional information relating to the statistical methods that may not be feasible to include in the main report.

Critical details of the trial adaptations (for example, the decision-making criteria or adaptation algorithm and rules) may be intentionally withheld from publicly accessible documents (for example, protocol) while the trial is ongoing.^45^ ^203^ These details may be documented in a formal document with restricted access and disclosed only when the trial is completed in order to minimise operational bias (item 11c). For this situation, authors should provide access to such details withheld with any amendments made for transparency and an audit trail of pre-planned AD aspects.

For some AD randomised trials, methods to derive statistical properties analytically may not be available. Thus, it becomes necessary to perform simulations under a wide range of plausible scenarios to investigate the operating characteristics of the design (item 7a), impact on estimation bias (item 12b), and appropriateness and consequences of decision-making criteria and rules.^154^ ^277^ In such cases, we encourage authors to reference accessible material used for this purpose (for example, simulation protocol and report, or published related material). Furthermore, it is good scientific practice to reference software, programs or code used for this task to facilitate reproducible research.

The operating characteristics of ADs heavily depend on following the pre-planned adaptations and adaptive decision-making criteria and rules. ADs often come with additional responsibilities for the traditional monitoring committees or require a specialised monitoring committee to provide independent oversight of the trial adaptations (for example, adaptive decision-making or adaptation committee). Thus, it is essential to be transparent about the adaptation decision-making process, roles and responsibilities of the delegated DMC(s), recommendations made by the committee and whether recommendations were adhered to. Authors are encouraged to provide supporting evidence (for example, DMC charter).

See box 27 for exemplars.

Box 27 **Exemplars on reporting item 24b**

*Example 1. Interim and final SAPs; IDMC roles and responsibilities; supplementary material*
  Léauté-Labrèze et al^98^ provide several versions of the SAP for a 2-stage inferentially seamless phase 2/3 AD as supplementary material. The remit and responsibilities of the IDMC including involvement in the adaptation decision-making process are detailed. The last version (3.5) of the SAP with amendments and details of interim and final analyses is found on pages 759 to 830 of the protocol supplementary material. Simulation results are summarised on pages 831 to 836.
*Example 2. Simulation report; supplementary material*
  Steg et al^181^ provide a simulation report evaluating the operating characteristics of a 3-arm 2-stage group sequential AD with dose selection under a number of scenarios in an appendix. The authors also explored the bias in methods used to estimate the treatment effects and confidence intervals and used the simulation results to inform their choice of methods.
*Example 3. Set-up of simulation studies and simulation results; published methodology work*
  Gu et al^208^ describe how simulation studies were performed and presented simulation results for evaluating operating characteristics of a 2-stage Bayesian biomarker-based AD.
*Example 4. Simulation report; published methodology work*
  Skrivanek et al^154^ published extensive simulation work quantifying operating characteristics of a Bayesian inferentially seamless phase 2/3 AD with RAR.
*Example 5. Simulation report; published methodology work*
  Heritier et al^67^ published extensive simulation work for an inferentially seamless phase 2/3 design using frequentist methods.

## Conclusions

There is a multidisciplinary desire to improve efficiency in the conduct of randomised trials. ADs allow pre-planned adaptations that offer opportunities to address research questions in randomised trials more efficiently compared to fixed designs. However, ADs can make the design, conduct and analysis of trials more complex. Potential biases can be introduced during the trial in several ways. Consequently, there are additional demands for transparency and reporting to enhance the credibility and interpretability of results from adaptive trials.

This CONSORT extension provides minimum essential reporting requirements that are applicable to pre-planned adaptations in AD randomised trials, designed and analysed using frequentist or Bayesian statistical methods. We have also given many exemplars of different types of ADs to help authors when using this extension. Our consensus process involved stakeholders from the public and private sectors.^13^ ^128^ We hope this extension will facilitate better reporting of randomised ADs and indirectly improve their design and conduct, as well as much-needed knowledge transfer.

**Abbreviations**

ACE, Adaptive designs CONSORT Extension
AD, adaptive design
CONSORT, Consolidated Standards Of Reporting Trials
E&E, explanation and elaboration
EQUATOR, Enhancing the QUAlity and Transparency Of health Research
(I)DMC, (independent) data monitoring committee
GSD, group sequential design
MAMS, multi-arm multi-stage design
MeSH, Medical Subject Heading
RAR, response-adaptive randomisation
SAP, statistical analysis plan
SSR, sample size re-estimation/re-assessment /re-calculation

## Web Extra

Extra material supplied by the author

Appendix A: Main ACE checklist available to download

Appendix B: Additional examples of Box 13

Appendix C. Example 2 of Box 14 (Bayesian RAR)

Appendix D: Example of a CONSORT flowchart for reporting 2-stage adaptive design (such as inferential seamless) that use combination test methods

Appendix E: Example of a CONSORT flowchart for reporting a population enrichment adaptive design (assuming enrichment was done at an interim analysis)

Appendix F: Example of a CONSORT flowchart for reporting a population enrichment adaptive design (assuming enrichment was not done at an interim analysis)

Appendix G: Example of a CONSORT flowchart for reporting a response-adaptive randomisation design with frequent randomisation updates

Appendix H: Example of a CONSORT flowchart for reporting a MAMS adaptive design

Appendix I: Box 22 - Dummy baseline table for the TAPPS trial

## Notes

Contributors: The idea originated from MD’s NIHR Doctoral Research Fellowship (DRF-2012-05-182) under the supervision of SAJ, ST, and JPN. The idea was presented, discussed, and contextualised at the 2016 annual workshop of the MRC Network of HTMR Adaptive Designs Working Group (ADWG), which was attended by six members of the Steering Committee (MD, TJ, PP, JW, APM, and CJW). MD, JW, TJ, APM, ST, SAJ, FK, CJW, DH, and JPN conceptualised the study design and applied for funding. All authors contributed to the conduct of the study and interpretation of the results. MD, ST, PP, CJW, JW, TJ and SAJ led the write-up of the manuscript. All authors except DGA contributed to the write-up of the manuscript, reviewed, and approved this final version. DGA oversaw the development process on behalf of the CONSORT Group and also contributed to the initial draft manuscript until his passing after the approval of the final checklists. The Steering Committee is deeply saddened by the passing of DGA who did not have the opportunity to approve the final manuscript. We dedicate this work to him in memory of his immense contribution to the ACE project, medical statistics, good scientific research practice and reporting, and humanity.

Members of the ACE Consensus Group contributed to the consensus workshop, approved the final ACE checklist, and were offered the opportunity to review the final manuscript.

Ethical approval and consent to participate: The project ethics approval was granted by the Research Ethics Committee of the School of Health and Related Research (ScHARR) at the University of Sheffield (ref: 012041). All Delphi participants during the development of the ACE guideline provided consent online during survey registration.

Dissemination: The results of the development process were disseminated to Delphi survey participants and the public.^13^ We will disseminate this guideline widely.
