# Supplementary material for: The Adaptive designs CONSORT Extension (ACE) statement: a checklist with explanation and elaboration guideline for reporting randomised trials that use an adaptive design
Source: BMJ. 2020 Jun 17;369:m115. doi: 10.1136/bmj.m115 (PMC7298567; doi:10.1136/bmj.m115)
Supplement: Supplementary file 1 — Appendix A: Main ACE checklist available to download [file dimm050350.w1.pdf]

Section/Topic	Item no	Checklist item	Page no
**Title and abstract**	1a	Identification as a randomised trial in the title	
	1b	Structured summary of trial design, methods, results, and conclusions (for specific guidance see ACE checklist for abstracts, Table 3)	
**Introduction**			
Background and objectives	2a	Scientific background and explanation of rationale	
	2b	Specific objectives or hypotheses	
**Methods**			
Trial design	3a	Description of trial design (such as parallel, factorial) including allocation ratio	
	3b« ǂ	Type of adaptive design used, with details of the pre-planned trial adaptations and the statistical information informing the adaptations	
	3c«3b ‡	Important changes to the design or methods after trial commencement (such as eligibility criteria) outside the scope of the pre-planned adaptive design features, with reasons	
Participants	4a	Eligibility criteria for participants	
	4b	Settings and locations where the data were collected	
Interventions	5	The interventions for each group with sufficient details to allow replication, including how and when they were actually administered	
Outcomes	6a ‡	Completely define pre-specified primary and secondary outcome measures, including how and when they were assessed. Any other outcome measures used to inform pre-planned adaptations should be described with the rationale	
	6b ‡	Any unplanned changes to trial outcomes after the trial commenced, with reasons	
Sample size and operating characteristics	7a ‡	How sample size and operating characteristics were determined	
	7b ‡‡	Pre-planned interim decision-making criteria to guide the trial adaptation process; whether decision-making criteria were binding or non-binding; pre-planned and actual timing and frequency of interim data looks to inform trial adaptations	
**Randomisation**			
Sequence generation	8a	Method used to generate the random allocation sequence	
	8b ‡	Type of randomisation; details of any restriction (such as blocking and block size); any changes to the allocation rule after trial adaptation decisions; any pre-planned allocation rule or algorithm to update randomisation with timing and frequency of updates	
Allocation concealment mechanism	9	Mechanism used to implement the random allocation sequence (such as sequentially numbered containers), describing any steps taken to conceal the sequence until interventions were assigned	
Implementation	10	Who generated the random allocation sequence, who enrolled participants, and who assigned participants to interventions	
Blinding	11a	If done, who was blinded after assignment to interventions (for example, participants, care providers, those assessing outcomes) and how	
	11b	If relevant, description of the similarity of interventions	
	11c ǂ	Measures to safeguard the confidentiality of interim information and minimise potential operational bias during the trial	
Statistical methods	12a ‡	Statistical methods used to compare groups for primary and secondary outcomes, and any other outcomes used to make pre-planned adaptations	
	12b« ǂ	For the implemented adaptive design features, statistical methods used to estimate treatment effects for key endpoints and to make inferences	
	12c«2b	Methods for additional analyses, such as subgroup analyses and adjusted analyses	
**Results**			
Participant flow (a diagram is strongly recommended)	13a ‡	For each group, the numbers of participants who were randomly assigned, received intended treatment, and were analysed for the primary outcome and any other outcomes used to inform pre-planned adaptations, if applicable	
	13b	For each group, losses and exclusions after randomisation, together with reasons	
Recruitment and adaptations	14a ‡	Dates defining the periods of recruitment and follow-up, for each group	
	14b †	Why the trial ended or was stopped	
	14c ǂ	Specify what trial adaptation decisions were made in light of the pre-planned decision-making criteria and observed accrued data	
Baseline data	15a«15 †	A table showing baseline demographic and clinical characteristics for each group	
	15b ǂ	Summary of data to enable the assessment of similarity in the trial population between interim stages	
Numbers analysed	16 †	For each group, number of participants (denominator) included in each analysis and whether the analysis was by original assigned groups	
Outcomes and estimation	17a †	For each primary and secondary outcome, results for each group, and the estimated effect size and its precision (such as 95% confidence interval)	
	17b	For binary outcomes, presentation of both absolute and relative effect sizes is recommended	
	17c ǂ	Report interim results used to inform interim decision-making	
Ancillary analyses	18	Results of any other analyses performed, including subgroup analyses and adjusted analyses, distinguishing pre-specified from exploratory	
Harms	19	All important harms or unintended effects in each group (for specific guidance see CONSORT for harms) [1]	
**Discussion**			
Limitations	20 †	Trial limitations, addressing sources of potential bias, imprecision, and, if relevant, multiplicity of analyses	
Generalisability	21 †	Generalisability (external validity, applicability) of the trial findings	
Interpretation	22	Interpretation consistent with results, balancing benefits and harms, and considering other relevant evidence	
**Other information**			
Registration	23	Registration number and name of trial registry	
Protocol	24a«24	Where the full trial protocol can be accessed	
SAP and other relevant trial documents	24b ǂ	Where the full statistical analysis plan and other relevant trial documents can be accessed	
Funding	25	Sources of funding and other support (such as supply of drugs), role of funders	

SAP, statistical analysis plan; ACE, Adaptive designs CONSORT Extension;
"X« Y" means original CONSORT 2010 item Y has been renumbered to X;
"X«" means item reordering resulted in new item X replacing the number of the original CONSORT 2010 item X
ǂ New items that should only be applied in reference to the ACE;
‡ Modified items that require reference to both CONSORT 2010 and ACE;
‡‡ Replacement (modified) item that only requires reference to the ACE;

† Item wording remains unchanged in reference to CONSORT 2010 but we expanded the ACE explanatory text to clarify additional considerations for certain adaptive designs. These unchanged items require reference to CONSORT 2010 except item 14b.
