# Supplementary material for: The Adaptive designs CONSORT Extension (ACE) statement: a checklist with explanation and elaboration guideline for reporting randomised trials that use an adaptive design
Source: BMJ. 2020 Jun 17;369:m115. doi: 10.1136/bmj.m115 (PMC7298567; doi:10.1136/bmj.m115)
Supplement: Supplementary file 2 — Appendix B: Additional examples of Box 13 [file dimm050350.w2.pdf]

Appendix B: Additional examples of Box 13

Box 13. Exemplars on reporting item 7b elements

Example 6. Sample size cap following SSR and treatment selection

"… the IDMC may recommend to increase the sample size at the interim analysis if there is less than 80% conditional power (CP) (*item 3b* details) to demonstrate superiority of the selected regimen (or at least one of the selected regimens in the case where two regimens are chosen) over placebo. The maximum permitted sample size increase will be fixed at 100 additional patients per selected propranolol regimen and 50 additional patients on the placebo arm"[1]. Extracted from supplementary material.

Example 7. 2-stage AD; use of non-binding futility boundary

"It has to be noted that, to protect the global type 1 error in case the decision was taken to overrule the futility rule, nonbinding boundaries would be used adding a very conservative boundary for efficacy. Overwhelming efficacy would be assessed on the adjudicated primary efficacy endpoint using the gamma (-10) alpha spending function[2] , in comparing the observed one-sided p-value with 0.00004 during this interim analysis. With such a conservative alpha spending function, the global alpha level of the study would be maintained at 0.05 (2-sided)."[3]. Extracted from supplementary material.

References

1 Léauté-Labrèze C, Hoeger P, Mazereeuw-Hautier J, *et al.* A Randomized, Controlled Trial of Oral Propranolol in Infantile Hemangioma. *N Engl J Med* 2015;**372**:735–46. doi:10.1056/NEJMoa1404710

2 Hwang IK, Shih WJ, De Cani JS. Group sequential designs using a family of type I error probability spending functions. *Stat Med* 1990;**9**:1439–45. doi:10.1002/sim.4780091207

3 Steg PG, Mehta SR, Pollack C V., *et al.* Anticoagulation with otamixaban and ischemic events in non-st-segment elevation acute coronary syndromes: The TAO randomized clinical trial. *JAMA* 2013;**310**:1145–55. doi:10.1001/jama.2013.277165
