# Supplementary material for: The Adaptive designs CONSORT Extension (ACE) statement: a checklist with explanation and elaboration guideline for reporting randomised trials that use an adaptive design
Source: BMJ. 2020 Jun 17;369:m115. doi: 10.1136/bmj.m115 (PMC7298567; doi:10.1136/bmj.m115)
Supplement: Supplementary file 3 — Appendix C. Example 2 of Box 14 (Bayesian RAR) [file dimm050350.w3.pdf]

Appendix C. Example 2 of Box 14 (Bayesian RAR)

"Patients were assigned to one of three treatment arms in an adaptive randomized fashion.[1] Initially, the randomization was balanced, with a probability of 1 in 3 of random assignment to each of the three arms. As data accrued about efficacy, assignment probabilities shifted in favour of arms that were performing better. The primary efficacy endpoint (success) was CR without nonhematologic grade 4 toxicity by 50 days. The comparison of arms in the data analysis and for the adaptive randomization was based on time to success, which we assumed was exponential, but which was truncated at 50 days. A priori, we assumed that the median time to success, m_i, for each treatment followed an inverse gamma (2.001, 4.614) distribution.

A maximum of 75 patients were to be randomized. Patients were to be randomly assigned to IA (arm 0), TA (arm 1), or TI (arm 2) with probabilities π_0, π_1, and π_2, respectively. Initially, $\pi_0 = \pi_1 = \pi_2 = \frac{1}{3}$. The probability of random assignment to IA (π_0) remained as 1/3, as long as all three arms remained in the trial. When each new patient entered the trial, q_k, which was defined as Pr($m_k < m_0$ data), where k=1,2; and r, defined as Pr($m_1 < m_2$ data), were calculated to evaluate the stopping rules and to adapt the randomization probabilities. Although all three treatments remained in the trial, the randomization probabilities, π_1, and $\pi_2$were calculated as $\pi_1 = \left(\frac{2}{3}\right)\frac{q_1^2}{q_1^2+q_2^2}$ and $\pi_2 = \left(\frac{2}{3}\right)\frac{q_2}{q_1^2+q_2^2}$ If at any time during the trial either q_1 >0.85 or q_2>0.85 (i.e., the current probability was at least 85% that TA or TI had a shorter time to CR than did IA), IA would be dropped from the randomization. If this were to happen and if both investigational arms were still in the trial, the randomization probability for arm 1, $\pi_1 = \frac{r^2}{r^2+(1-r^2)}$, and the probability of assignment to arm 2 would become $\pi_2 = 1 - \pi_1$. If at any time q_1 <0.15 (i.e., TA was being outperformed by IA) or r <0.15 (i.e., TA was being outperformed by TI), TA would be dropped from randomization. In addition, if q_2 <0.15 (TI was being outperformed by IA) or if r >0.85 (TI was being outperformed by TA), TI would be dropped from randomization. If at any time during the trial only IA and one investigational arm k remained, the randomization probability of arm q was set to $\pi_k = \frac{q_k^2}{q_k^2+(1-q_k^2)}$ and the randomization probability for the control was set to $\pi_0 = 1 - \pi_k$. Finally, an arm that dropped out could be reopened if information (i.e., CR by day 49) became available from patients previously randomly assigned to that arm or if the other arms performed sufficiently poorly, subsequent to closure of the arm in question"[2]

References

1 Berry DA, Eick SG. Adaptive assignment versus balanced randomization in clinical trials: A decision analysis. *Stat Med* 1995;**14**:231–46. doi:10.1002/sim.4780140302

2 Giles FJ, Kantarjian HM, Cortes JE, *et al.* Adaptive Randomized Study of Idarubicin and Cytarabine Versus Troxacitabine and Cytarabine Versus Troxacitabine and Idarubicin in Untreated Patients 50 Years or Older With Adverse Karyotype Acute Myeloid Leukemia. *J Clin Oncol* 2003;**21**:1722–7. doi:10.1200/JCO.2003.11.016
