# Supplementary material for: The Adaptive designs CONSORT Extension (ACE) statement: a checklist with explanation and elaboration guideline for reporting randomised trials that use an adaptive design
Source: BMJ. 2020 Jun 17;369:m115. doi: 10.1136/bmj.m115 (PMC7298567; doi:10.1136/bmj.m115)
Supplement: Supplementary file 4 — Appendix D: Example of a CONSORT flowchart for reporting 2-stage adaptive design (such as inferential seamless) that use combination test methods [file dimm050350.w4.pdf]

Appendix D: An example of a CONSORT flowchart for reporting a 2-stage adaptive design (e.g. inferential seamless) that use combination test methods

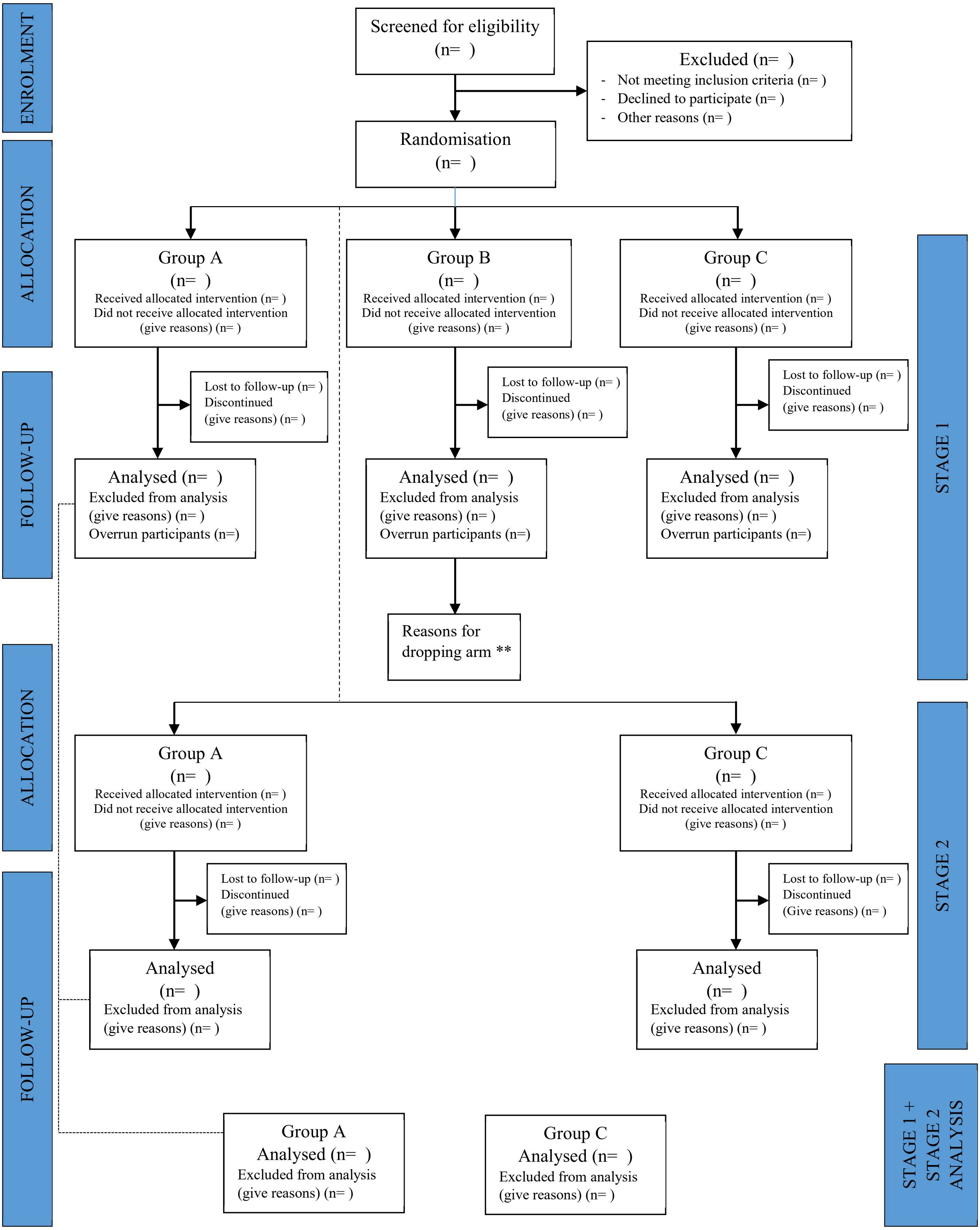

** This can be extended to reflect any further analysis that was performed after an interim analysis when a treatment arm was stopped (e.g. to include overrun participants who did not contribute to the interim analysis or long-term outcome data)
