# Supplementary material for: The Adaptive designs CONSORT Extension (ACE) statement: a checklist with explanation and elaboration guideline for reporting randomised trials that use an adaptive design
Source: BMJ. 2020 Jun 17;369:m115. doi: 10.1136/bmj.m115 (PMC7298567; doi:10.1136/bmj.m115)
Supplement: Supplementary file 5 — Appendix E: Example of a CONSORT flowchart for reporting a population enrichment adaptive design (assuming enrichment was done at an interim analysis) [file dimm050350.w5.pdf]

Appendix E: An example of a CONSORT flowchart for reporting a population enrichment adaptive design (assuming enrichment was done at an interim analysis)

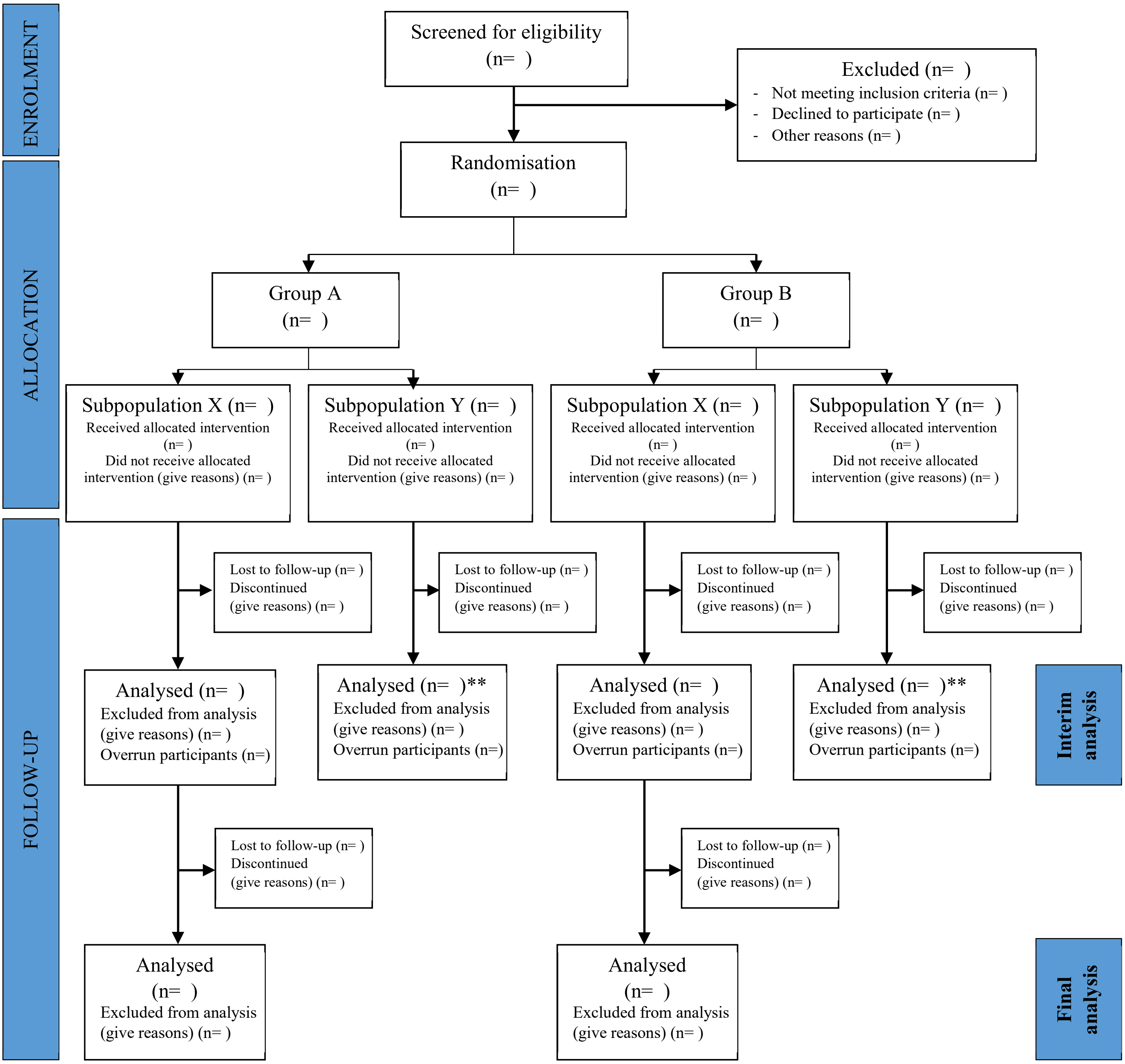

** This can be extended to the final analysis if an additional analysis was performed after an interim analysis when further recruitment to a specific subpopulation was stopped (e.g. to include overrun participants who did not contribute to the interim analysis or long-term outcome data)
