# Supplementary material for: The Adaptive designs CONSORT Extension (ACE) statement: a checklist with explanation and elaboration guideline for reporting randomised trials that use an adaptive design
Source: BMJ. 2020 Jun 17;369:m115. doi: 10.1136/bmj.m115 (PMC7298567; doi:10.1136/bmj.m115)

Appendix F: An example of a CONSORT flowchart for reporting a population enrichment adaptive design (assuming enrichment was not done at an interim analysis)

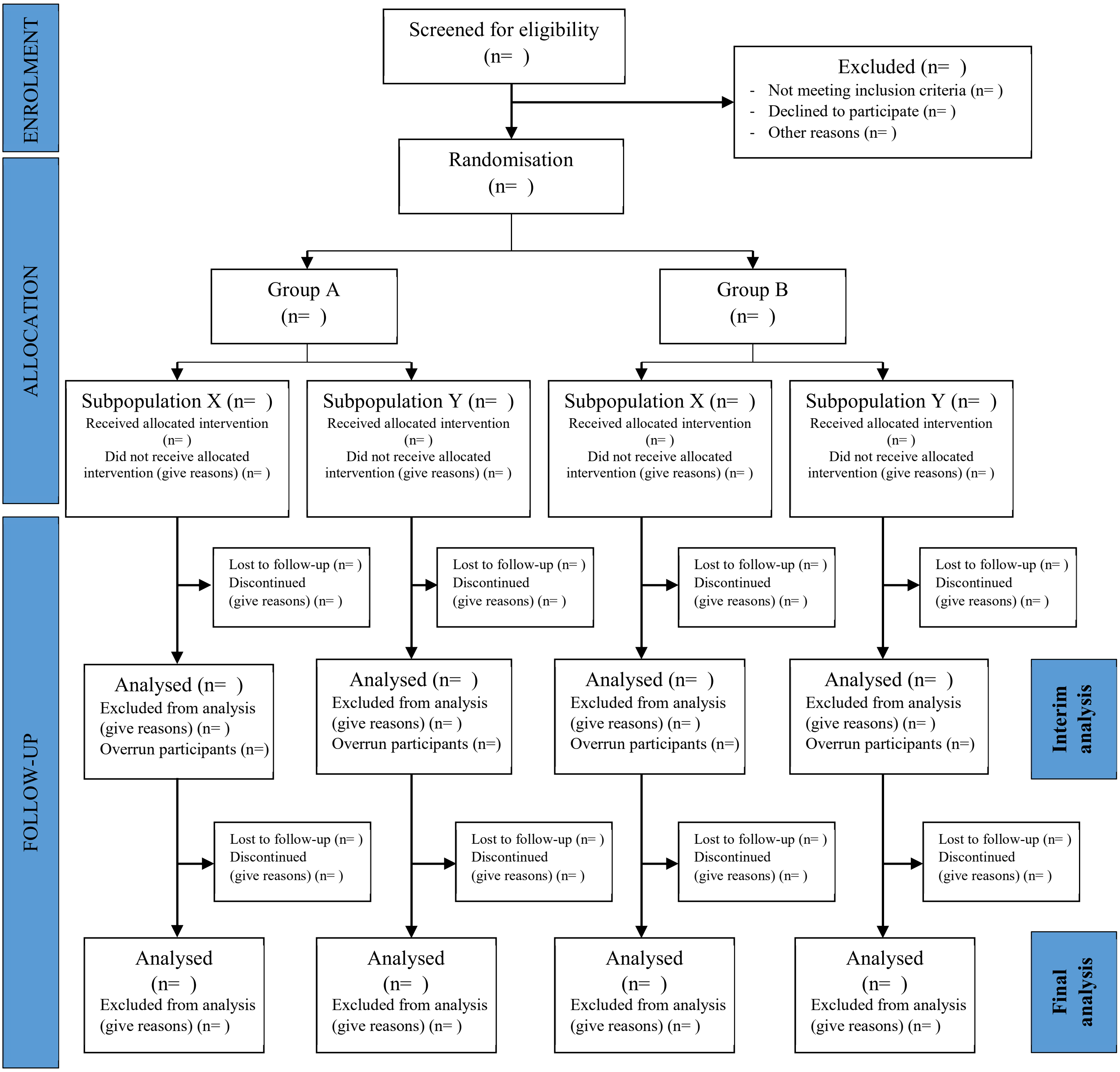

Supplement: Supplementary file 6 — Appendix F: Example of a CONSORT flowchart for reporting a population enrichment adaptive design (assuming enrichment was not done at an interim analysis) [file dimm050350.w6.pdf]
