# Supplementary material for: The Adaptive designs CONSORT Extension (ACE) statement: a checklist with explanation and elaboration guideline for reporting randomised trials that use an adaptive design
Source: BMJ. 2020 Jun 17;369:m115. doi: 10.1136/bmj.m115 (PMC7298567; doi:10.1136/bmj.m115)
Supplement: Supplementary file 8 — Appendix H: Example of a CONSORT flowchart for reporting a MAMS adaptive design [file dimm050350.w8.pdf]

Appendix H: An example of a CONSORT flowchart for reporting a MAMS adaptive design

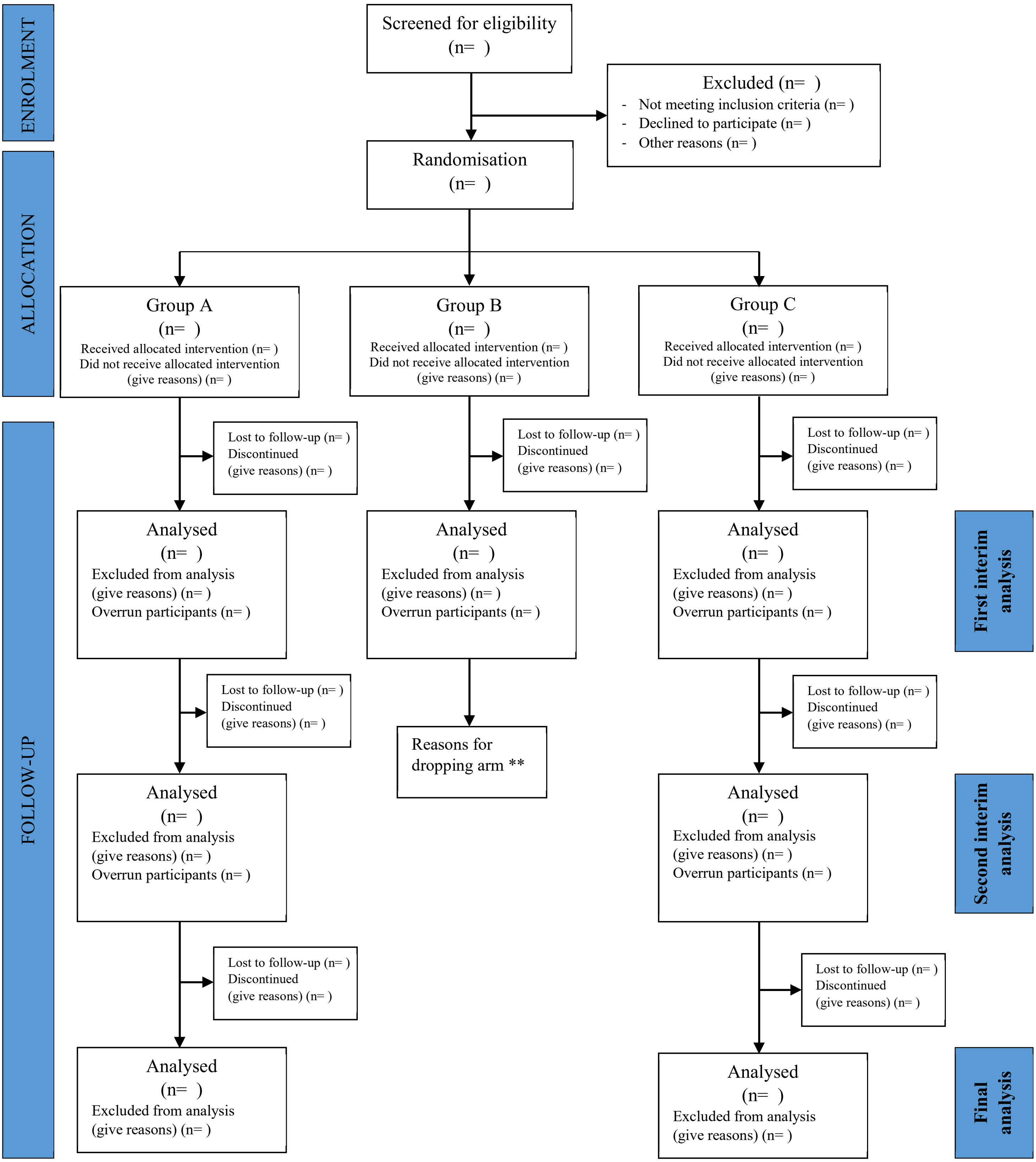

** This can be extended to the final analysis if an additional analysis was performed after an interim analysis when a treatment arm was stopped (e.g. to include overrun participants who did not contribute to the interim analysis or long-term outcome data)
