# Supplementary material for: The Adaptive designs CONSORT Extension (ACE) statement: a checklist with explanation and elaboration guideline for reporting randomised trials that use an adaptive design
Source: BMJ. 2020 Jun 17;369:m115. doi: 10.1136/bmj.m115 (PMC7298567; doi:10.1136/bmj.m115)
Supplement: Supplementary file 9 — Appendix I: Box 22 - Dummy baseline table for the TAPPS trial [file dimm050350.w9.pdf]

Appendix I: Box 22 - Dummy baseline table for the TAPPS trial

Characteristic	Full population (cutaneous and non-cutaneous)		Subpopulation (cutaneous)	
	TRC105 and Pazopanib (n=xx)	Pazopanid alone (n=xx)	TRC105 and Pazopanib (n=xx)	Pazopanid (n=xx)
Age, years				
Mean(SD)	xx(xx.x)	xx(xx.x)	xx(xx.x)	xx(xx.x)
Min, max	xx, xx	xx, xx	xx, xx	xx, xx
Sex, n(%)				
Female	xx(xx%)	xx(xx%)	xx(xx%)	xx(xx%)
Male	xx(xx%)	xx(xx%)	xx(xx%)	xx(xx%)
Height, m				
Mean(SD)	xx(xx.x)	xx(xx.x)	xx(xx.x)	xx(xx.x)
Min, max	xx, xx	xx, xx	xx, xx	xx, xx
Weight, Kg				
Mean(SD)	xx(xx.x)	xx(xx.x)	xx(xxx)	xx(xxx)
Min, max	xx, xx	xx, xx	xx, xx	xx, xx
Plasma Levels:				
VEGF[1]				
Mean(SD)	xx(xx.x)	xx(xx.x)	xx(xx.x)	xx(xx.x)
Min, max	xx, xx	xx, xx	xx, xx	xx, xx
sVEGFR2[2]				
Mean(SD)	xx(xx.x)	xx(xx.x)	xx(xx.x)	xx(xx.x)
Min, max	xx, xx	xx, xx	xx, xx	xx, xx
sCD105[3]				
Mean(SD)	xx(xx.x)	xx(xx.x)	xx(xx.x)	xx(xx.x)
Min, max	xx, xx	xx, xx	xx, xx	xx, xx
PIGF[4]				
Mean(SD)	xx(xx.x)	xx(xx.x)	xx(xx.x)	xx(xx.x)
Min, max	xx, xx	xx, xx	xx, xx	xx, xx
CTCs[5]				
Mean(SD)	xx(xx.x)	xx(xx.x)	xx(xx.x)	xx(xx.x)
Min, max	xx, xx	xx, xx	xx, xx	xx, xx

[1] vascular endothelial growth factor; [2] soluble vascular endothelial growth receptor; [3] soluble endoglin; [4] placental growth factor; [5] numbers of endoglin expressing circulating tumor cells; min, minimum; max, maximum; SD, standard deviation, Kg, kilograms; m, metres
